# A conserved fungal glycosyltransferase facilitates pathogenesis of plants by enabling hyphal growth on solid surfaces

**DOI:** 10.1371/journal.ppat.1006672

**Published:** 2017-10-11

**Authors:** Robert King, Martin Urban, Rebecca P. Lauder, Nichola Hawkins, Matthew Evans, Amy Plummer, Kirstie Halsey, Alison Lovegrove, Kim Hammond-Kosack, Jason J. Rudd

**Affiliations:** 1 Department of Computational and Analytical Sciences, Rothamsted Research, Harpenden, Herts, United Kingdom; 2 Wheat Pathogenomics Team, Department of BioInteractions and Crop Protection, Rothamsted Research, Harpenden, Herts, United Kingdom; 3 Rothamsted Centre for Bioimaging, Department of Plant Sciences, Rothamsted Research, Harpenden, Herts, United Kingdom; 4 Fungicide resistance group, Department of BioInteractions and Crop Protection, Rothamsted Research, Harpenden, Herts, United Kingdom; 5 Cereal cell walls group, Department of Plant Sciences, Rothamsted Research, Harpenden, Herts, United Kingdom; Institute of Microbiology, CHINA

## Abstract

Pathogenic fungi must extend filamentous hyphae across solid surfaces to cause diseases of plants. However, the full inventory of genes which support this is incomplete and many may be currently concealed due to their essentiality for the hyphal growth form. During a random T-DNA mutagenesis screen performed on the pleomorphic wheat (*Triticum aestivum*) pathogen *Zymoseptoria tritici*, we acquired a mutant unable to extend hyphae specifically when on solid surfaces. In contrast “yeast-like” growth, and all other growth forms, were unaffected. The inability to extend surface hyphae resulted in a complete loss of virulence on plants. The affected gene encoded a predicted type 2 glycosyltransferase (ZtGT2). Analysis of >800 genomes from taxonomically diverse fungi highlighted a generally widespread, but discontinuous, distribution of *ZtGT2* orthologues, and a complete absence of any similar proteins in non-filamentous ascomycete yeasts. Deletion mutants of the *ZtGT2* orthologue in the taxonomically un-related fungus *Fusarium graminearum* were also severely impaired in hyphal growth and non-pathogenic on wheat ears. *ZtGT2* expression increased during filamentous growth and electron microscopy on deletion mutants (ΔZtGT2) suggested the protein functions to maintain the outermost surface of the fungal cell wall. Despite this, adhesion to leaf surfaces was unaffected in ΔZtGT2 mutants and global RNAseq-based gene expression profiling highlighted that surface-sensing and protein secretion was also largely unaffected. However, ΔZtGT2 mutants constitutively overexpressed several transmembrane and secreted proteins, including an important LysM-domain chitin-binding virulence effector, *Zt3LysM*. ZtGT2 likely functions in the synthesis of a currently unknown, potentially minor but widespread, extracellular or outer cell wall polysaccharide which plays a key role in facilitating many interactions between plants and fungi by enabling hyphal growth on solid matrices.

## Author summary

All plant-pathogenic fungi must grow hyphae across host tissues and cells to establish diseases. We have identified a single glycosyltransferase enzyme from the pleomorphic wheat pathogen *Zymoseptoria tritici* which functions specifically to enable hyphal growth on solid surfaces, and is therefore essential for fungal disease of wheat plants. *ZtGT2* orthologues are present in most ascomycete filamentous fungi, and we show that the orthologous gene from the distantly related wheat ear infecting fungus, *Fusarium graminearum*, is also required for hyphal growth and virulence. Conversely the gene is completely absent from the genomes of most ascomycete yeast species, which do not form true hyphae. The data suggests that *ZtGT2* orthologues may have played an important role in the evolution of pathogenic fungi, by enabling hyphal growth on solid surfaces. It is likely that this capability, which is also a requirement for the establishment of mutualistic interactions and for saprophytic growth, arose “inadvertently” from the need for fungi to adopt the characteristic filamentous lifestyle which enables them to seek out and access distal nutrient sources.

## Introduction

Micro-organisms have evolved many different mechanisms to enable them to cause diseases of plants. Some of these mechanisms are pathogen species-host plant specific. For example, the evolution and deployment of suites of secreted “effector” proteins, allow pathogens to manipulate components of plant immunity to support infection [1]. The molecular interplay which underpins this exquisite control of host-pathogen interactions has become the focus of considerable research aimed at improving disease resistance in crop plants [2].

However, prior to engaging fully with plant immunity, fungal pathogens must first adhere to, recognise, respond to, and then grow on or through plant surfaces to initiate infection [3]. All known fungi infecting the aerial tissues of plants (flowers, leaves, stems) are referred to as “filamentous”, meaning that although they arrive on plant surfaces as either air- or water-borne spores, they subsequently transition into filamentous hyphae to grow over or through tissues and cells [4]. This discriminates filamentous fungi from the model ascomycete yeast species *Saccharomyces cerevisiae* and *Schizosaccharomyces pombe* and their close relatives, which are free living and do not form true hyphae. Many fungi which are pathogenic on animals can switch between yeast and hyphal growth forms (dimorphic), but most frequently in these cases the infectious module are often the spores, resulting in “yeast-like” infections [5–7]. In contrast, almost all (if not all) major plant infecting fungi use hyphal growth to initiate and/or complete infection. Therefore, hyphal growth over solid surfaces is a pre-requisite step for potentially all fungal diseases of plants.

Fungal hyphae are structurally supported by complex outer cell walls, which have multiple layered components of proteins and sugars and provide the strength and flexibility which enable growth, and sensing of growth surfaces [8, 9]. Major structural components of fungal cell walls include the complex polysaccharides chitin and beta glucans, which are present in most species. Both of these polysaccharides are also known to be triggers of plant defences, via their recognition by plant plasma membrane immune receptors [10]. However, fungal cell walls also contain various other polysaccharides including alpha glucans [11, 12] and other structurally undefined, but likely important molecules. Moreover, as fungi grow across and through solid surfaces they are also likely to deploy some form of extracellular matrix (ECM) to counteract surface-derived frictional and shear stresses [13, 14]. The importance of ECM is widely recognised for mammalian cells, which produce a matrix which contains a complex linear polysaccharide called Hyaluronan, as a key component [15, 16]. In contrast, whilst the presence of ECM surrounding fungal cells and hyphae has been detected via various staining and microscopy methods [17, 18], the precise polysaccharide components therein have only in rare cases been structurally defined or functionally characterised [19, 20]

The ascomycete filamentous fungus *Zymoseptoria tritici* (class Dothideomycete) is the causal agent of Septoria tritici blotch (STB) disease of wheat leaves, which represents one of the most economically important crop diseases of wheat worldwide [21, 22]. *Z*. *tritici* is also regarded as an emerging model fungus, due in part to its ability to grow in several different morphological states (or forms) depending on environmental conditions [23–25]. For this reason, *Z*. *tritici* is referred to as a pleomorphic fungus [26], able to grow in at least two different growth forms. One advantage of experimenting with pleomorphic (or dimorphic) fungi is that genes which are essential for life (or the recovery of stably transformed gene deletion strains) for only one growth form can still be characterised in viable cells growing in the alternative growth form(s). It is more problematic to define the functions of putative essential genes in fungi with only a single dominant growth form. Consistent with most plant pathogenic fungi, *Z*. *tritici* infection of wheat leaves begins with spores alighting, either through wind or rain splash, onto leaf surfaces. Initial infection then requires the spores to germinate and grow hyphae across the leaf surface and into stomatal pores [25, 27]. Once inside leaves, *Z*. *tritici* secretes a plethora of important effector proteins during hyphal growth [25], including a broadly conserved Lysin domain (LysM) effector protein which masks fungal chitin from plant immune receptors [28–32]. It is currently unknown what factors can trigger the up-regulation of effector production during phases of leaf infection.

This current study derives from a forward’s genetic screen aimed to identify novel virulence factors from *Z*. *tritici*, which contribute to its ability to cause plant disease. The data we present reports identification of a widely conserved but discontinuously distributed glycosyltransferase which plays a crucial role in pathogenicity of not only *Z*. *tritici*, but also a distinct and taxonomically un-related pathogen of wheat, *Fusarium graminearum*. We demonstrate its key role in enabling pathogenicity is to allow hyphal filaments to extend over solid surfaces. The distribution of orthologous genes within the fungal kingdom, suggests that this role in virulence likely evolved “inadvertently” from a more generic need for fungi to scavenge for nutrients through extending hyphal filaments over solid surfaces. The current study also identified an unexpected role for this protein in the regulation of pathogen effector gene expression.

## Results and discussion

### Isolation of a non-pathogenic *Z*. *tritici* T-DNA mutant unable to extend hyphae on solid surfaces

**Fig 1:**
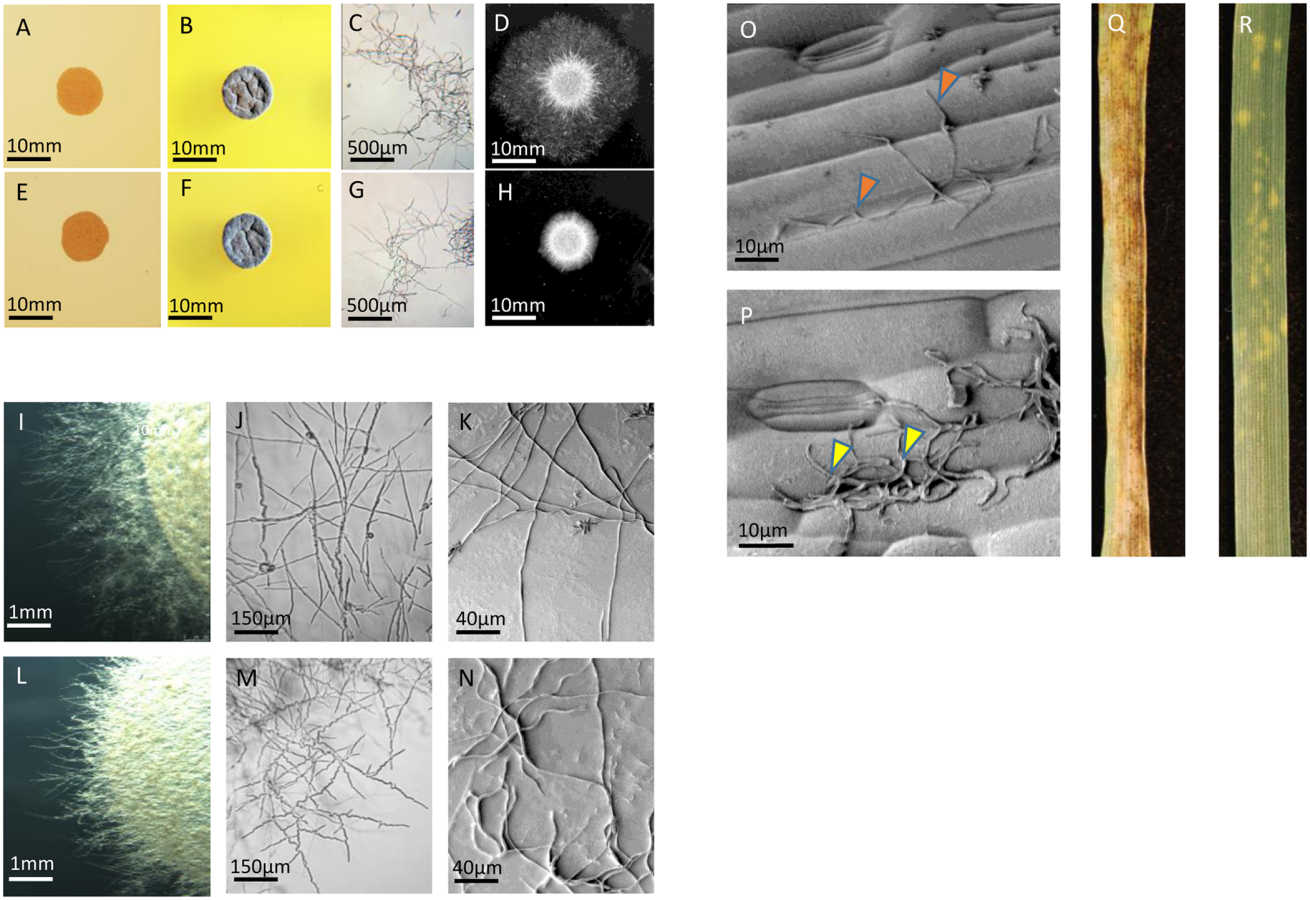
Characterisation of a non-pathogenic T-DNA mutant of *Z*. *tritici* (23–170) unable to extend hyphae on solid surfaces. (A-D) Typical growth characteristics of wild type (WT) *Z*. *tritici* on rich nutrient agar at 16°C (A); rich agar at 25°C (B); sterile liquid water (C) and solid water agar (D). (E-H) display the comparable growth morphologies of mutant 23–170. Note the severely impaired hyphal growth from the colony on solid water agar (H). (I-K) micrographs displaying hyphal morphology of wild type *Z*. *tritici* on solid water agar. (L-N) comparable micrographs displaying morphology of short, sinusoidal hyphae formed by 23–170. (O) SEM analysis of wild type fungal inoculated wheat leaf surfaces at 48 hpi. Arrows highlight surface growing hyphae. (P) SEM of 23–170 mutant cells at 48hpi on wheat leaf surfaces. Arrows highlight un-germinated fungal spores. (Q) Wheat leaf disease symptoms photographed 14 days after inoculation with wild type fungus. (R) Comparable levels of leaf disease caused by the 23–170 mutant at 14 dpi.

*Z*. *tritici* can grow in a “yeast-like” budding form or extend true hyphal filaments on agar plates depending on temperature and nutritional status. Fig 1 displays the growth phenotypes of the wild type (WT) fungus on different nutrient agar plates, at different temperatures, and in sterile liquid water. At 16°C on solid nutrient-rich Yeast extract peptone dextrose (YPD) agar, WT *Z*. *tritici* spores undergo yeast-like budding generating a mass of pink coloured spores (Fig 1A). On the same plates incubated at 25°C the fungus subsequently forms extensive networks of melanised aerial hypha over the top of the colony, with no direct contact with the underlying agar (Fig 1B). In nutrient limiting sterile shaking water flasks, spores germinate to form hyphal filaments in suspension (Fig 1C) and on solid water agar plates at 25°C spores germinate hyphae which extend radially from the spore droplet across the surrounding agar (1D). Fig 1E to 1H display the corresponding growth morphologies of a transposable DNA (T-DNA) tagged mutant of *Z*. *tritici* called 23–170 under the same conditions. The mutant had identical growth under all conditions (Fig 1E to 1G) except for growth on solid water agar, where hyphal growth was severely impaired (Fig 1H). Yeast-like growth of 23–170 on YPD agar occurred at a rate comparable to the WT strain and spore morphologies were macroscopically indistinguishable (S1 Fig). The filamentous growth defect of 23–170 on solid water agar was also seen at all tested agar strengths (S2 Fig) and during growth on Czapek-Dox and PDA agar, each of which can also support some level of filamentous growth by *Z*. *tritici* after protracted incubations (S3 Fig). Closer inspection of the hyphal filaments of both WT and 23–170 on water agar by both light microscopy and scanning electron microscopy (SEM) revealed that, in contrast to the mostly long and straight filaments produced by the WT strain (Fig 1L and 1K), the 23–170 filaments were both shorter and grew in a sinusoidal (“wavy”) manner (Fig 1L–1N). These data suggested that the mutation(s) present in 23–170 rendered it incapable of normally extending hyphae when in contact with a solid surface. Significantly, this phenotype was supported by wheat leaf infection data. In contrast to the WT strain which could rapidly elaborate hyphal filaments on the surfaces of wheat leaves (Fig 1O) and subsequently cause full disease (Fig 1Q), the 23–170 mutant displayed no clear hyphal growth on leaf surfaces (Fig 1P). This resulted in a dramatic loss of disease causing ability (Fig 1R). On occasions, some limited chlorosis was observed on leaves inoculated with 23–170, but these never progressed into necrotic lesions and no fungal sporulation was ever observed (Fig 1R).

### The 23–170 mutant phenotype results from inactivation of a gene encoding a predicted type 2 glycosyltransferase

**Fig 2:**
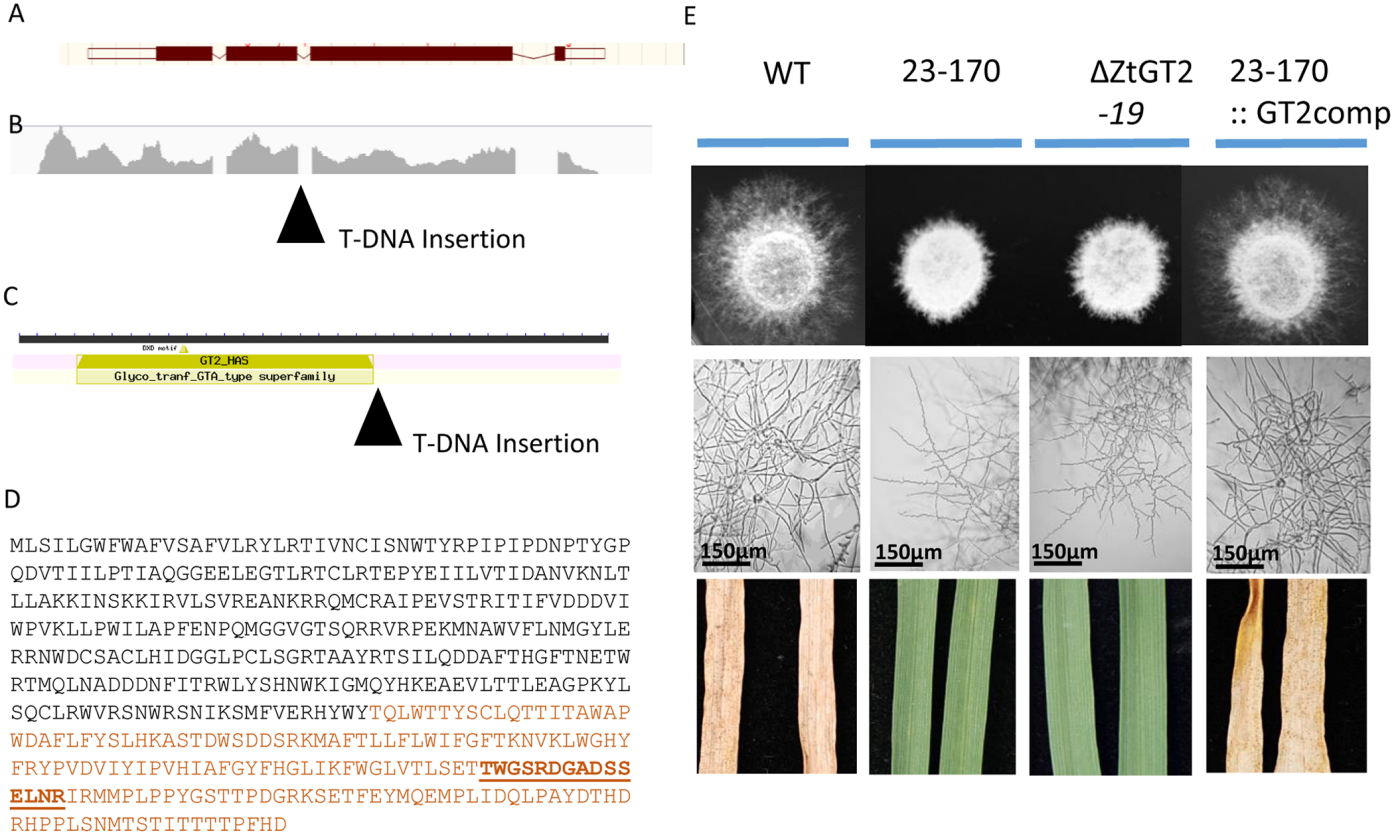
All mutant phenotypes of 23–170 result from inactivation of a gene encoding a putative type 2 glycosyltransferase (ZtGT2). (A) The gene model structure identified through TAIL-PCR analysis of the T-DNA insertion site in mutant 23–170. (B) RNAseq raw read mapping confirmed the predicted gene structure and highlights the position of the left border T-DNA insertion. (C) Functional annotation of the tagged gene highlighting the position of the T-DNA left border relative to the predicted type 2 glycosyltransferase catalytic domain. (D) The predicted amino acid sequence of ZtGT2 highlighting the protein region truncated by T-DNA insertion in brown font. The underlined region indicates the peptide sequence chosen for antibody generation (E) Complementation of the 23–170 mutant strain with the wild-type *ZtGT2* gene (23–170::GT2comp) restores hyphal growth on solid surfaces and virulence on plants. Independent targeted deletion of *ZtGT2* in the wild-type fungus (ΔZtGT2-19) results in the same aberrant hyphal growth and loss of virulence phenotypes shown for 23–170.

Thermal asymmetric interlaced (TAIL)-PCR was used to identify the T-DNA insertion site in 23–170, with recovered sequences then subjected to a Blastn analysis against the fully sequenced genome of strain IPO323 [33] (http://genome.jgi.doe.gov/pages/search-for-genes.jsf?organism=Mycgr3). All sequenced PCR products mapped an identical T-DNA insertion site within the second intron of a gene model present on the antisense strand of Chromosome 1, between nucleotide positions 1786483-1788643 (Fig 2A). This gene model was well supported by RNA sequencing (RNAseq) raw read mapping data (Fig 2B), and the gene was annotated as encoding a type 2 glycosyltransferase (GT2) represented by Genbank ID XP_003857553.1 (Fig 2C). The T-DNA insertion likely resulted in a severely truncated protein (Fig 2D). To validate whether loss of the *Z*. *tritici* GT2 (ZtGT2) function was responsible for all the phenotypes seen for 23–170, the wild-type gene plus upstream and downstream regions was transformed back into strain 23–170 for complementation analysis. In addition, the *ZtGT2* gene was also subject to independent targeted deletion in the WT fungus. Full hyphal growth on water agar and plant disease causing ability were restored by complementation of 23–170 with the native gene, and the targeted ΔZtGT2 mutants displayed all the original 23–170 mutant phenotypes (Fig 2D) including short “wavy” hyphal formation on the surface of all tested agar plates (S4 Fig). Thus, we conclude that the ZtGT2 protein plays an essential role in supporting the virulence of *Z*. *tritici* through enabling hyphal growth over solid surfaces.

### Orthologues of *ZtGT2* are widely but discontinuously distributed within the fungal kingdom

**Fig 3:**
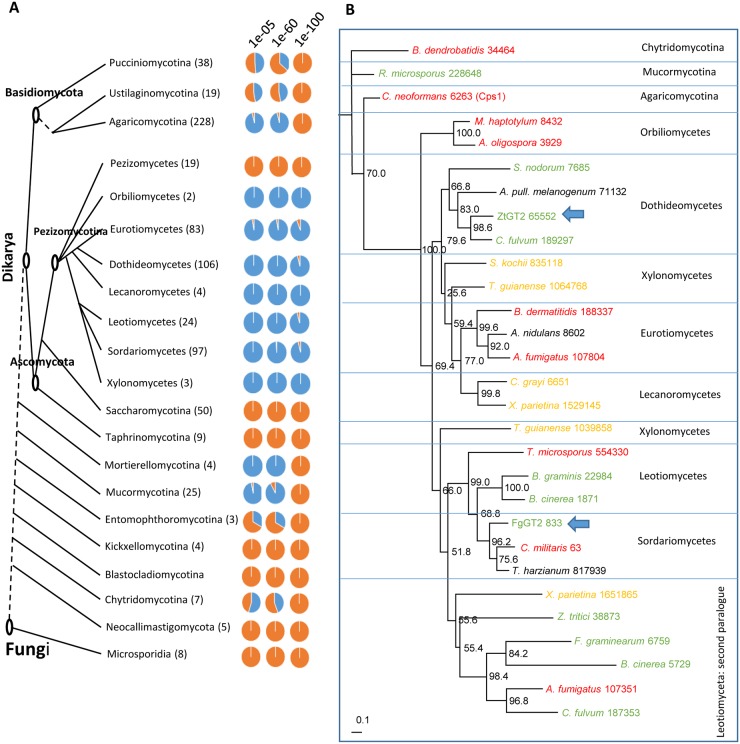
*ZtGT2* orthologues show a discontinuous distribution throughout the fungal tree of life but are present in saprophytes, mutualists and pathogens. (A) Diagrammatic representation of the fungal tree of life adapted from the JGI MycoCosm genome portal (http://genome.jgi.doe.gov/programs/fungi/index.jsf). Taxonomic tree distances are only representative and do not accurately indicate evolutionary time. Each fungal taxonomic class or sub phylum is shown with brackets highlighting the number of species genomes present in each group analysed. Pie charts then display the number of species genomes which have a at least one similar protein to ZtGT2 at each of the indicated Blastp expect thresholds. Blue shading indicates the number of species with at least one similar proteins. Orange shading indicates the number of species with no similar proteins. (B) Maximum Likelihood phylogenetic tree displaying representative sequence orthologues to *ZtGT2* from across the fungal tree of life. Node labels indicate percentage bootstrap support (500 replicates). Where similar genes were detected orthology is inferred by correspondence to the fungal species tree. Species names in green font indicate known plant pathogens, those in red font indicate known animal pathogens and those in yellow indicate those with mutualistic lifestyles. A true saprophytic lifestyle is included in the form of *Aspergillus nidulans* and other species in black font have currently ambiguous lifestyles. Numbers following species names represent the protein model Id retrievable from the JGI Mycocosm species genome portal.

A Blastp analysis using the mature ZtGT2 protein sequence at expect (e-value) cut-off thresholds of 1.0e-100, 1.0e-60 and 1.0e-5 was performed on the predicted proteomes of 823 fungi present at the JGI Mycocosm portal [34] representing 20 different taxonomic classes or sub phylum (Fig 3A and S1 Table). Overall, proteins with varying degrees of similarity to ZtGT2, were identified in many of the species analysed including some of the earliest evolved fungal groups (Fig 3A and S1 Table). Phylogenetic analysis on a subset of transcripts representative of taxonomically distinct higher (ascomycete and basidiomycetes) and lower fungi, with parasitic, mutualistic or saprotrophic lifestyles (S2 Table), confirmed that orthologues of *ZtGT2* were found in accordance with the fungal species tree and were present in some lower fungi (eg Chytrids and Mucor species) as well as many, but not all, basidiomycetes (Fig 3B). In contrast, most filamentous ascomycete fungi possess an orthologue of *ZtGT2* irrespective of whether they were parasitic, mutualistic or saprotrophic. Numerous independent duplications were detected within basidiomycete species, but within ascomycetes, the 2^nd^ Leotiomyceta paralogues grouped together, suggesting one duplication event there. (Fig 3B and S1 Table). A single paralogue of *ZtGT2* was identified in *Z*. *tritici* (protein model 38873 in Fig 3B). The dramatic phenotypes observed for ΔZtGT2 mutants suggest this paralogue does not have an overlapping functionality. Interestingly no ascomycete species belonging to the class Pezizomycetes possessed orthologues, or any similar proteins whatsoever, to ZtGT2, highlighting a discontinuous distribution within this kingdom most likely arising from gene loss (Fig 3A and S1 Table). This distribution was further emphasised by the fact that no orthologues (or proteins with any similarity even at Blastp e-5) were identified in the genomes (proteomes) of all 59 ascomycete yeasts including all tested members of the Saccharomycotina and Taphrinomycotina, which include the model species *Saccharomyces cerevisiae*, *Schizosaccharomyces pombe* and the yeast-like human pathogenic *Candida* species (Fig 3A and S1 Table). The discontinuous distribution of proteins with similarity to ZtGT2 was also very evident within the tested Basidiomycetes (Fig 3A and S1 Table) with orthologues most frequently observed in the Agaricomycotina, represented by the gene model 6263 (Cps1) from *Cryptococcus neoformans* in Fig 3B.

### The orthologous gene from the unrelated ascomycete wheat pathogen *Fusarium graminearum* is also important for hyphal growth and virulence on plants

**Fig 4:**
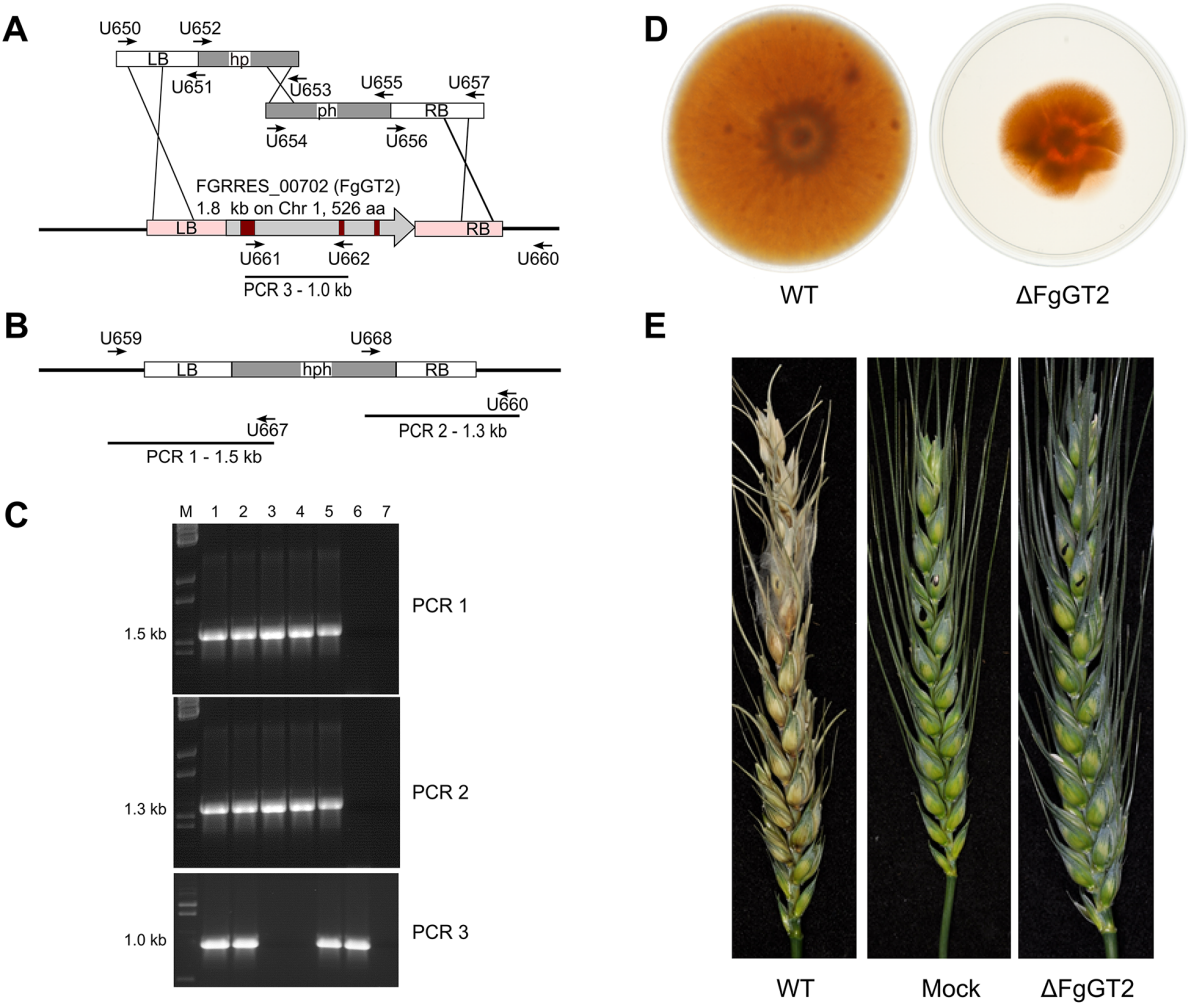
The *ZtGT2* orthologue from the wheat ear blight pathogen *Fusarium graminearum* (*FgGT2*) is required for hyphal growth on solid surfaces and virulence. The *F*. *graminearum* gene *FGRRES_00702* (http://fungi.ensembl.org/Fusarium_graminearum/Info/Index) orthologous to *ZtGT2* was functionally characterized by gene deletion using a selection step in liquid rather than solid medium. (A) Genomic left border and right border regions (white bars) were amplified with primers and fused to parts of the *hph* hygromycin resistance gene. Fused PCR fragments were used in a split-marker strategy to replace FGRRES_00702. Horizontal black bars represent genomic areas outside the replacement construct, vertical black bars represent 3 introns. (B) Anticipated diagnostic PCR for successful gene replacement of FGRRES_00702. (C) Results of diagnostic PCR and expected sizes indicated in (A) and (B). Loadings are: M—λ DNA-BstEII digest, 1–5 transformants MU424 to MU428, WT, no DNA. MU426 and MU427 have the ΔFGRRES_00702 null allele and lost the 1.0 kb GT2 fragment. (D) Severely reduced growth for the *FgGT2* null allele mutants on the surface of potato dextrose agar plates after 8 days incubation compared to the wild-type PH-1 strain. (E) Wheat ears inoculated with *FgGT2* null mutant and wild-type strain 13 days post-inoculation. Spore-droplet inoculated spikelets are marked with black dots. For the mock inoculation, only water was used.

To test whether orthologues of ZtGT2 conferred the same function in other ascomycete fungi, we attempted to generate deletion strains in the wheat ear pathogen, *Fusarium graminearum*, a member of the taxonomically distant Sordariomycete class of fungi (Fig 3A and highlighted in Fig 3B). The gene selected for testing was *FG00702*.*1* (http://fungi.ensembl.org/Fusarium_graminearum/Info/Index) with Blastp homology of 2.74e-142 to ZtGT2, encompassing 51% amino acid identity spanning 78.9% of the protein model. Phylogenetic analysis on transcripts confirmed the likely 1 to 1 orthology between ZtGT2 and FgGT2 (Fig 3B). *F*. *graminearum* is a rapidly growing filamentous fungus which unlike *Z*. *tritici* exhibits no known yeast-like growth form. Using standard protocols which select for hygromycin resistant transformants on solid agar [35], we were unable to recover any homokaryotic *FgGT2* deletion strains, but instead recovered numerous heterokaryons. PCR analysis on these strains demonstrated that they possessed both a wild-type allele in addition to a deleted allele of *FgGT2*, and they grew hyphae at normal rates on agar plates (S5 Fig). It is known that attempts to delete “essential” genes in multinucleate fungi places strong selective pressure on the formation of such heterokaryons [36]. In view of this, and the phenotype described for ΔZtGT2 mutants, we modified the transformation protocol to perform selection in liquid medium instead of on solid agar (see Methods). This approach led to the acquisition of two independent homokaryotic deletion strains of *FgGT2* (Fig 4C). Significantly both mutant strains were severely impaired in radial hyphal growth on solid agar (Fig 4D) and completely impaired in virulence towards wheat ears when either point inoculated via droplet, or sprayed across the entire wheat ear (Fig 4E). In addition to the radial growth defect seen on the surface of agar, the ΔFgGT2 mutants were also tested for ability to grow into (penetrate) agar plates through cellophane sheets [37]. This revealed that mutants were still capable of this type of invasive growth. However, this assay again demonstrated that subsequent radial growth on the agar surface was strongly affected (S6 Fig). This data highlights that invasive growth, which represents a key additional feature of *F*. *graminearum* infection of plants not known to occur in *Z*. *tritici*, does not require functional FgGT2 (S6 Fig).

### *ZtGT2* transcripts accumulate during hyphal growth and mutant cells display outer cell wall abnormalities

**Fig 5:**
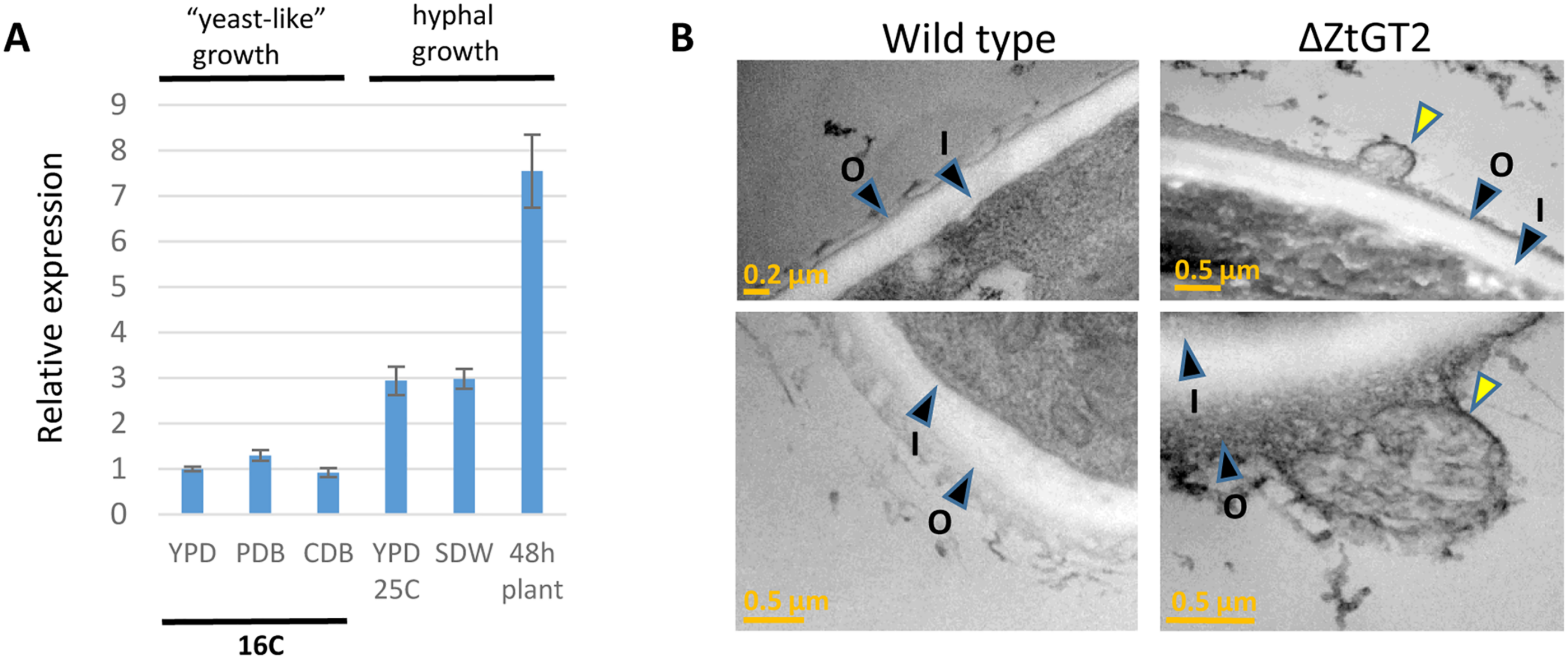
*ZtGT2* transcripts accumulate under conditions which stimulate hyphal growth and mutant cells display an abnormal outer cell wall structure. (A) Real-Time qPCR analysis on *ZtGT2* expression. Overall expression is relatively low in three different liquid culture media (Yeast extract peptone dextrose broth (YPD); Potato dextrose broth (PDB) and Czapek-Dox (CDB) broth) grown at 16°C. Transcript levels are increased during growth in YPD at 25°C and during growth in liquid water (24h) which both stimulate more hyphal growth. Highest transcript accumulation overall was observed during hyphal growth on wheat leaf surfaces at 48 hpi. Data illustrate the results of three replicate experiments with standard error bars deriving from the combined data. (B) Transmission electron micrographs display abnormal structures on the outer cell wall of ΔZtGT2 mutants (highlighted with yellow arrowhead). These structures were observed only in ΔZtGT2 mutants and seen in ~50% of all sections analysed. The outer (O) and inner (I) cell wall regions are highlighted with black arrowheads.

Quantitative Real-Time PCR (qPCR) gene expression profiling demonstrated that *ZtGT2* was expressed most strongly under conditions which favour hyphal growth of *Z*. *tritici*, including elevated temperatures, low nutrients and early (48h) growth on wheat leaf surfaces (Fig 5A). To ascertain the likely protein localisation of ZtGT2, we generated a peptide antibody. Western analysis demonstrated that this antibody cross reacted with a specific ~52kDa protein present in wild type cells but absent from ΔZtGT2 mutants (S7 Fig). This protein was detected in the low speed cell wall pellet of fungal cells extracted in detergent-free buffer. In contrast, no protein was present in the soluble fraction, and little to none in the high speed microsomal fraction. This suggested that ZtGT2 may be present in, or attached to, the fungal cell wall (S7 Fig).

Analysis of cell wall ultrastructure by TEM, following collection of yeast-like spores from the surfaces of solid agar plates, revealed the frequent presence of outer wall surface irregularities, which appeared as bulges, protrusions or as breakages (Fig 5B and S8 Fig). These were observed in over 50% of all sections examined from mutant cells and were never seen in sections of WT cells, suggesting that ZtGT2 plays a role in generating or maintaining an outer wall component. We then sought to determine whether there were any differences in monosaccharide levels (glucose, mannose and galactose) of the alcohol insoluble polysaccharides deriving from either the cell wall or culture filtrates of ΔZtGT2 relative to WT strains. We were unable to determine any quantitative differences in these monosaccharides deriving from total cell walls (S9A Fig). Comparable analysis of the ethanol precipitated culture filtrates revealed an unexpected increase in monosaccharides from ΔZtGT2 relative to WT (S9B Fig). Thus, overall the results were inconclusive but we detected no depletion of materials due to loss of ZtGT2. This may suggest that the product of ZtGT2 is a relatively minor component of either the fungal cell wall or extracellular matrix. In this case its overall contribution to the total monosaccharide composition may be masked by the more abundant chitin, beta- and alpha- glucans. It is also possible that loss of ΔZtGT2 function could induce changes, such as the alterations seen in outer cell wall structure (Fig 5B and S8 Fig) that could result in / from changes in the levels of other polysaccharides, influenced indirectly by loss of ZtGT2 function.

We also developed assays to determine whether the outer wall irregularities gave rise to changes in the ability of conidia to adhere to leaf surfaces (S10 Fig), or to plastic (hydrophobic) surfaces (S10 Fig). Like most fungi which are pathogenic on leaves of plants, the spores of *Z*. *tritici* adhere very strongly and rapidly to hydrophobic surfaces such as those posed by waxy leaf surfaces [3]. No change in the ability of ΔZtGT2 mutant spores to adhere to either of these hydrophobic surfaces was detected (S10 Fig) demonstrating that the macroscopic differences observed between the outer walls of each strain did not compromise surface attachment. Similarly, ΔZtGT2 mutant spores displayed no altered sensitivity to chemical de-stabilisation of chitin synthesis (Calcoflour white), beta glucan function (Caspofungin), temperature (30°C), oxidative (H_2_O_2_) or osmotic (sorbitol) stress (S11 Fig), suggesting that major cell wall and membrane components were not strongly affected by gene loss.

### ZtΔGT2 mutants retain ability to respond to plant surfaces, but constitutively overexpress chitin-binding effectors and transmembrane proteins

**Fig 6:**
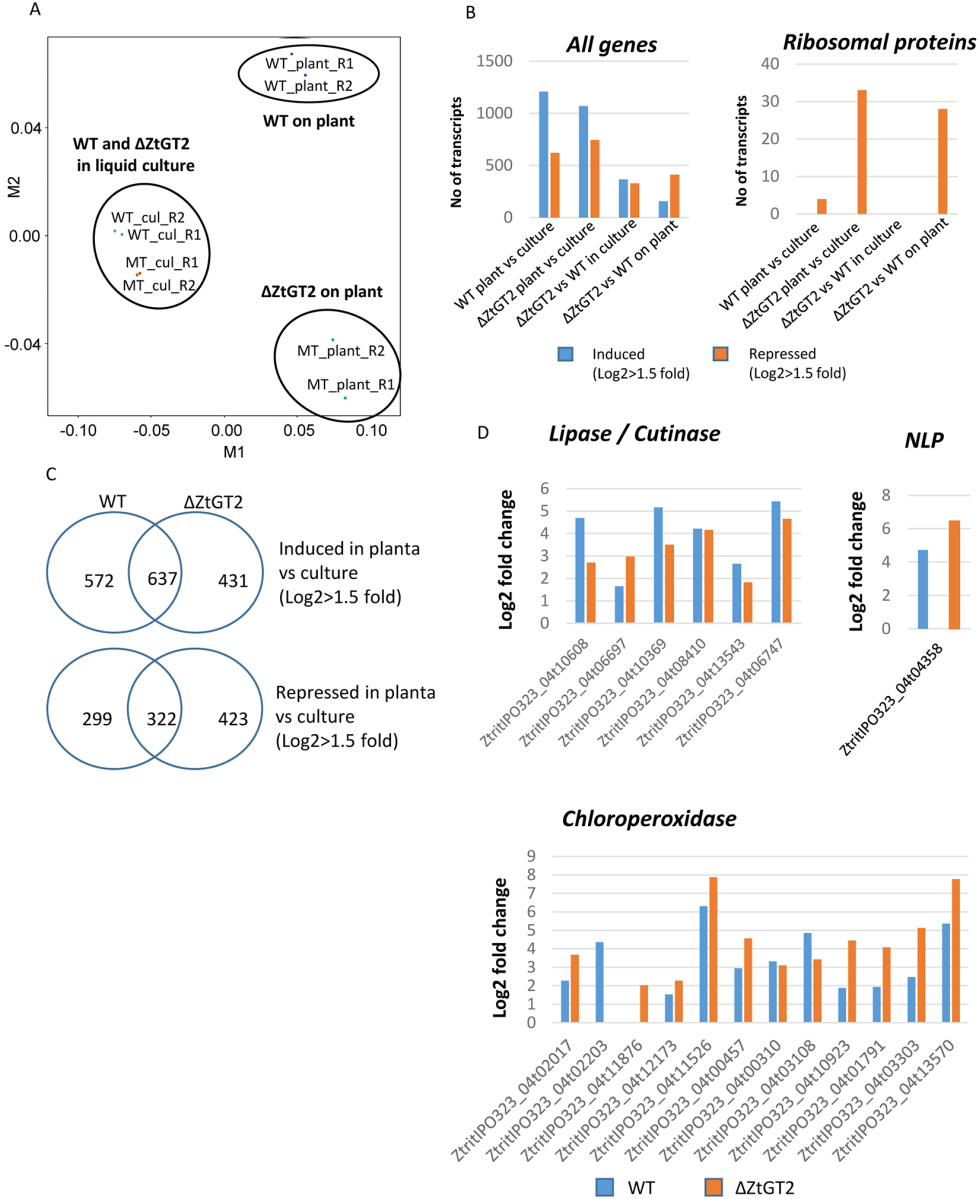
Global RNAseq expression profiling highlights that ΔZtGT2 mutants are rapidly arrested on leaf surfaces but still retain many early transcriptional responses. (A) Global clustering analysis of fungal RNAseq data from growth of wild type and ΔZtGT2 mutants in liquid culture, and at 48h after leaf inoculation. Key to data point labelling- WT_plant_R1 indicates wild type fungus on plant surface 1^st^ replication; MT_cul_R2 indicates ΔZtGT2 mutant in YPD culture broth 2^nd^ replicate sample. (B) Rapid arrest of growth on wheat leaf surfaces is indicated by dramatically reduced expression of many genes encoding ribosomal protein in ΔZtGT2. (C) Many genes induced and repressed by the WT fungus following leaf inoculation retain the same dynamics in ΔZtGT2 mutants. (D) Many classes of early expressed genes encoding secreted proteins are up-regulated to equivalent levels in both the WT and ΔZtGT2 mutant strains indicating surface sensing is still retained. All data is expressed relative to expression levels detected in the equivalent fungal liquid culture. (NLP = necrosis and ethylene inducing protein).

Based upon the abnormalities seen on the outer wall surfaces of ΔZtGT2 mutants, and their failure to germinate hyphae on leaf surfaces, we speculated that leaf surface-sensing may be compromised in the mutants. To test this, we performed RNAseq based whole genome expression profiling of early leaf infection compared with growth in liquid culture. Materials were sampled as shown in S12 Fig to allow several pairwise analyses on the fungal and plant transcriptomes. Global Principle Components Analysis (PCA) on the fungal datasets highlighted that, relative to growth of WT and ΔZtGT2 strains in liquid culture, 48 h growth on leaf surfaces distinguished their respective transcriptomes the most (Fig 6A and S3 Table). Much of this effect was attributed to relative growth rates on wheat leaf surfaces, as illustrated by the repression of many ribosomal protein encoding genes in ΔZtGT2 on the leaf surface. This feature was not observed when comparing the two strains transcriptomes in liquid culture, which further emphasised the key role of ZtGT2 in regulating contact-dependent fungal growth (Fig 6B). Despite this, many genes normally expressed by the WT fungus on leaves relative to liquid culture, were also similarly expressed by ΔZtGT2 (Fig 6C and S3 Table). These included the characteristic early transcriptional up-regulation of suites of secreted protein encoding genes including *Lipases*, *Cutinases*, *Necrosis-inducing proteins* (*NLP*) and *Chloroperoxidases* [24, 38–40] (Fig 6D). These data highlight that whilst hyphal growth is rapidly impaired, ΔZtGT2 can still sense and react transcriptionally to the leaf surface environment. The fungal leaf infection data also highlighted several protein families which are transcriptionally upregulated by the WT fungus on leaves, but which are not by ΔZtGT2. One example being several CFEM domain containing membrane-spanning proteins [41] which were strongly negatively affected in ΔZtGT2, both in liquid culture and even more so on leaf surfaces (S13 Fig). This data suggests these genes may play key roles in regulating processes associated with elongating hyphae during normal plant infection by the WT fungus.

The most strongly up-regulated genes in ΔZtGT2 growing in liquid culture encoded secreted or membrane bound proteins. Table 1 highlights that amongst the “top 30” genes upregulated in the ΔZtGT2 mutant relative to WT, twenty-eight fell into these categories. Seventeen of these 28 were also upregulated in the WT fungus at 48h on leaf surfaces relative to its growth in liquid culture (Table 1). This suggested that loss of ZtGT2 function stimulated some transcriptional changes to occur which are commonly induced early by the fungus on wheat leaf surfaces. Amongst this set of genes was one encoding a functionally validated effector protein, *Zt3LysM* (Gene Id ZtritIPO323_04t03143 in Table 1 and S14 and S15 Figs), which binds to chitin fragments and mediates the evasion of plant chitin-triggered immunity [31, 32]. This suggests that the loss of normal outer cell wall structure in ΔZtGT2 mutants may mimic changes which usually occur following inoculation onto plants, and that this may serve to trigger increased expression of the *Zt3LysM* effector.

**Table 1:** The Top30 most upregulated genes in ΔZtGT2 vs WT fungus growing in liquid medium.

*Gene_id new Ensembl	**Original Id Ensembl	FPKM WT	FPKM ΔZtGT2	Log2 fold change ΔZtGT2/WT	Blastp annotation (NCBI)	Signal peptide?	Transmembrane?	log2 fold WT 48h plant / culture
ZtritIPO323_04t13800	Mycgr3G47776	2.38361	187.623	6.29855	GPR1 FUN34 -class plasma membrane	No	YES	3.69719
ZtritIPO323_04t06606	Mycgr3G103427	36.5046	2328.37	5.9951	Tandem Repeat Protein 4 (TRP4)***	Yes	No	4.56836
ZtritIPO323_04t08955	Mycgr3G109137	50.7599	2372.23	5.54641	hypothetical protein MYCGRDRAFT_109137	Yes	No	4.48632
**ZtritIPO323_04t03143**	**Mycgr3G111221**	**21.2326**	**742.174**	**5.12741**	**3xLysM domain effector (3LysM)**	**Yes**	**No**	**5.30617**
ZtritIPO323_04t10723	Mycgr3G104794	327.83	10093.8	4.94438	hypothetical protein MYCGRDRAFT_104794	Yes	No	1.90893
ZtritIPO323_04t01868	Mycgr3G89984	9.07987	277.305	4.93266	hypothetical protein MYCGRDRAFT_89984	No	YES	2.02453
ZtritIPO323_04t07933	Mycgr3G91855	5.51628	168.164	4.93003	aspergillopepsin-2 precursor like	Yes	No	No change
ZtritIPO323_04t01956	Mycgr3G67250	4.68437	124.825	4.73591	peroxidase catalase like	Yes	No	1.71448
ZtritIPO323_04t00231	Mycgr3G34306	5.01835	132.068	4.71792	Aspartic protease pep1 like	Yes	No	3.47866
ZtritIPO323_04t13637	Mycgr3G95831	20.0937	519.83	4.69323	hypothetical protein MYCGRDRAFT_95831	Yes	No	No change
ZtritIPO323_04t04294	Mycgr3G111636	235.826	5976.16	4.66342	Ecp2-3 effector homologue	Yes	No	1.9105
ZtritIPO323_04t08501	Mycgr3G104000	38.9405	950.309	4.60905	hypothetical protein MYCGRDRAFT_104000	Yes	No	6.39787
**ZtritIPO323_04t10384**	**Mycgr3G72646**	**6.27375**	**148.704**	**4.56697**	**alpha 1,3 glucan synthase**	**No**	**YES**	**1.76247**
ZtritIPO323_04t11777	Mycgr3G110220	32.942	745.202	4.49963	hypothetical protein MYCGRDRAFT_110220	Yes	No	2.4987
ZtritIPO323_04t04149	Mycgr3G77689	2.1705	43.3503	4.31994	carboxypeptidase S1	Yes	No	5.99527
ZtritIPO323_04t03806	Mycgr3G97031	19.0535	376.75	4.30548	hypothetical protein MYCGRDRAFT_97031	Yes	No	No change
ZtritIPO323_04t09707	none	28.0532	550.981	4.29576	none	Yes	No	No change
ZtritIPO323_04t13185	Mycgr3G48129	91.2925	1728.88	4.2432	Cerato-platanin / hydrophobin	Yes	No	No change
ZtritIPO323_04t11297	Mycgr3G12051	1.65444	28.5554	4.10935	galactose oxidase	Yes	No	4.3809
ZtritIPO323_04t09426	Mycgr3G71659	4.53293	77.3318	4.09255	NAD(P)-binding Rossmann-fold containing	No	No	No change
ZtritIPO323_04t07171	Mycgr3G108399	38.5037	644.32	4.06471	hypothetical protein MYCGRDRAFT_108399	No	No	No change
ZtritIPO323_04t03262	Mycgr3G96651	1.87684	31.0977	4.05043	hypothetical protein MYCGRDRAFT_96651	No	No	No change
ZtritIPO323_04t04165	none	9.77584	156.07	3.99683	hypothetical protein TI39_contig4278g00011	Yes	No	5.34383
ZtritIPO323_04t00584	none	77.6554	1221.01	3.97484	hypothetical protein TI39_contig279g00027	No	No	No change
ZtritIPO323_04t09555	Mycgr3G92998	25.6961	402.709	3.97012	hypothetical protein MYCGRDRAFT_92998	Yes	No	1.87831
ZtritIPO323_04t06766	Mycgr3G69117	4.18014	64.4432	3.9464	MFS monosaccharide transporter like	No	YES	No change
ZtritIPO323_04t11928	Mycgr3G19274	257.455	3908.59	3.92425	major allergen alt a1	Yes	No	No change
ZtritIPO323_04t13136	Mycgr3G46937	8.05712	121.448	3.91393	major facilitator superfamily transporter like	No	YES	No change
ZtritIPO323_04t12624	Mycgr3G105419	276.522	4164.69	3.91274	small threonine-rich	Yes	No	-1.58721
ZtritIPO323_04t13161	Mycgr3G101121	2.95145	42.827	3.85902	general substrate transporter	No	YES	5.83776

*- sequences available from https://doi.org/10.6084/m9.figshare.4753708.v1 [42].

** sequences available from http://fungi.ensembl.org/Zymoseptoria_tritici/Info/Index.

*** previously described in [43].

Also notable amongst the genes shown in Table 1 was a strong up-regulation (>25 fold levels in the WT strain) of a transcript predicted to encode an alpha-1,3-glucan synthase in the ΔZtGT2 mutant (ZtritIPO323_04t10384 in Table 1). In the filamentous fungal pathogen of animals *Aspergillus fumigatus*, alpha-1,3-glucan represents a major constituent of the outer cell wall and is required for full virulence [11, 12]. In contrast to the putative *Z*. *tritici alpha-1*,*3-glucan synthase* gene, no other genes likely to be involved in the biosynthesis of either chitin or beta-glucans were differentially expressed in the mutant relative to the WT. Hence from the data here provided it may be that loss of the outer wall integrity in ΔZtGT2 triggers a specific compensatory activation of alpha glucan synthesis.

Analysis of the wheat leaf transcriptome at 48h hpi with ΔZtGT2 and the WT fungus also revealed many differentially expressed genes (S4 Table). Prominent amongst these were plant defence-associated genes including those encoding pathogenesis-related (PR) proteins, receptor-like kinases (RLK) and WRKY transcription factors, which were all more highly expressed in leaves infected with the WT fungus than those infected with ΔZtGT2 (S16 Fig). This likely reflects the comparable lack of hyphal growth by the ΔZtGT2 mutants, relative to that by the WT fungus, over the growth period. In support of this, independent qPCR assessments on wheat defence gene expression incorporating mock-inoculated leaves (no fungus) indicated that the lower induction of plant defence genes by ΔZtGT2 was unlikely to be a consequence of loss of a specific elicitor activity (S16 Fig).

## Conclusions

All fungal pathogens of plants require the ability to grow hyphae over solid surfaces posed by host tissues and cells to initiate or complete infections. The present study identified a widely (but not universally) conserved glycosyltransferase (GT2) as a likely key facilitator of this process. The pleomorphic growth characteristics of *Zymoseptoria tritci* enabled the identification of the specific growth state in which ZtGT2 plays a crucial role, that being hyphal growth when in physical contact with solid matrices. It is likely for fungi which exhibit a predominantly hyphal growth state on solid agar, that gene deletion mutants of *ZtGT2* orthologues may be difficult to recover using standard protocols. This could be interpreted as either them being “essential” genes in filamentous fungi, or that hyphal growth is so severely affected in mutants that they cannot be recovered. In support of the latter interpretation was our requirement to perform selection in a liquid, rather than solid growth medium, to obtain homokaryotic ΔFgGT2 deletion mutants in the highly filamentous fungus, *Fusarium graminearum*. This highlights that the terminology “essential gene” should be used with caution and in consideration of the technical approaches used, when interpreting failed attempts to obtain specific gene deletion mutants from filamentous fungi. In contrast, the fact that yeast-like growth of *Z*. *tritici* ΔZtGT2 mutants occurs un-impeded on solid agar, highlights the utility of this species for characterising genes which may be “essential” for hyphal growth in fungi. The ability for *Z*. *tritici* to undergo “yeast-like” budding growth in the absence of *ZtGT2*, agrees well with the observation that all true free-living ascomycete budding yeasts, particularly in the classes Saccharomycotina and Taphrinomycotina, lack any similar proteins. This suggests that ZtGT2 orthologues may be dispensable for the budding growth form of ascomycete fungi.

The basidiomycete pathogen of mammals, *Cryptococcus neoformans*, is a dimorphic fungus pathogenic in the yeast form, but which can also grow as true hyphae during sexual reproduction [44]. *C*. *neoformans* possesses a ZtGT2 orthologue named CPS1 (Blastp homology of 9.05e-90 to ZtGT2 with 47.3% identity covering 73.5% of the protein), shown in Fig 3B. This protein is predicted to function as a putative capsule polysaccharide synthase and is essential for full virulence of the yeast form against mammals [45, 46]. In contrast to this, the yeast-like non-pathogenic growth form of *Z*. *tritici* is unaffected in ΔZtGT2 mutants, and it is instead the infectious hyphal growth form which is affected, and then only when in physical contact with surfaces. This re-emphasises a key distinguishing feature of infection of plants and animals by ascomycete and basidiomycete fungi which can replicate in either yeast-like or filamentous forms. The polysaccharide produced by CPS1 has been suggested to be similar to Hyaluronic acid, the key and ubiquitous extracellular matrix component of mammals, buts it’s precise structure has not yet been determined. The morphological abnormalities we detected in the ΔZtGT2 mutant suggests that ZtGT2 plays a key role in maintaining outer cell wall integrity, although there is currently no evidence to suggest that *Z*. *tritici* (or any filamentous fungus), forms anything functionally equivalent to the unique *C*. *neoformans* yeast capsule [47]. Efforts to detect quantitative changes in cell wall or culture filtrate monosaccharides (deriving from precipitated polysaccharide) between WT and ΔZtGT2 mutants of *Z*. *tritici* were inconsistent. Neither of the analyses performed identified decreased levels of monosaccharides deriving from polysaccharides from ΔZtGT2 mutants. For total cell walls, the levels of glucose, mannose and galactose were unchanged. Conversely the culture filtrate of ΔZtGT2 mutants unexpectedly gave increased levels. As fungal cell walls possess chitin and beta glucan as some major components, it is possible that other functionally important, but more minor constituents might be beyond detection if only using comparisons between gene deletion strains with wild-type strains. It is also possible that such components may have been lost in preparation without prior knowledge of their physicochemical properties. However, identifying differences via the approach of comparing WT strains with gene deletion mutants in this case was also likely complicated by the fact that our transcriptome data demonstrated that ΔZtGT2 strains overexpress a conserved alpha-1,3- glucan synthase to ~25 fold higher levels than WT strains (Table 1). So its possible that the increased levels of polysaccharides derived from culture filtrates of ΔZtGT2 mutants might be a consequence of other polysaccharides being over-produced to compensate for the loss of ZtGT2. It may also be that the irregularities seen in the outer cell wall surface may have also contributed to the increased levels of monosaccharides detected. This requires further study. However, future approaches aimed to identify and structurally characterise the polysaccharide produced by ZtGT2 and/or its orthologues may be better addressed using different approaches to circumvent these potential complications.

**Fig 7:**
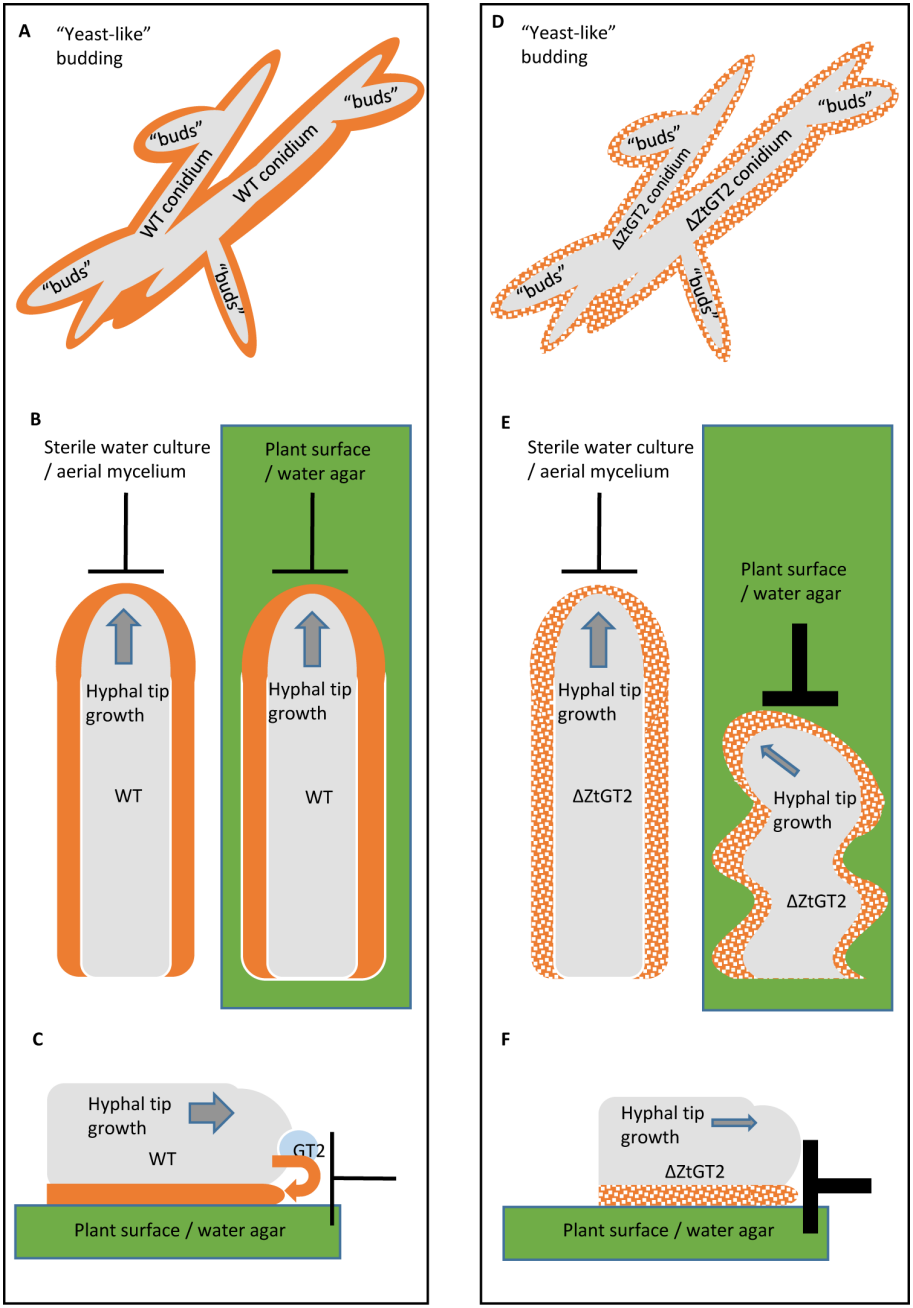
Model for the function of ZtGT2 in overcoming surface-derived frictional (opposing) forces. The phenotype of ΔZtGT2 mutants, the predicted protein localisation, homology to *C*. *neoformans* polysaccharide synthase CPS1, and alterations to the outer cell wall, suggest that ZtGT2 is involved in the production of an unknown polysaccharide which acts as an important component of the outer cell wall or extracellular matrix (ECM) of fungal cells (indicated by solid orange colouring surrounding grey cells in wild type fungus depicted in A to C). The cells missing this component are depicted by hatched shading surrounding ΔZtGT2 mutants in D to F. In budding spores (conidia) of *Z*. *tritici* this component is functionally dispensable and both wild type and ΔZtGT2 mutants grow at comparable rates (A and D). However, for *Z*. *tritici* hyphae this feature may provide the fluidity which enables rapid extension in a forward’s direction in the face of the counter frictional forces of the leaf (or other) surfaces (B and C). These counter forces resulting from frictional or other stresses are depicted as cross hatches throughout (B, C, E and F). ΔZtGT2 lacks a functional version of the outer cell wall, or ECM, polysaccharide so whilst mutant cells can grow normally as hyphae in liquid suspensions (where they encounter no frictional counter forces- left panel of E) they are unable to effectively counter these forces on solid surfaces (right panel of E and F). This gives rise to short, occasionally sinusoidal (or aborted) hyphae on agar and a loss of virulence on plants.

At this point it is unclear why some filamentous fungi lack orthologues, or proteins with any similarity whatsoever, to ZtGT2. For example, the model basidiomycete plant pathogens *Puccinia graminis* and *Ustilago maydis* have no similar proteins to ZtGT2, but they do grow hyphae over plant surfaces (including wheat leaves for the former). As presence / absence of proteins with similarity to ZtGT2 appears discontinuous, even within the ascomycete kingdom, it is possible that some fungal species use a different protein, or mechanism, to provide the equivalent functionality. This might again be reflected in the chemical composition of the outer cell wall surfaces in these fungi. However, for ZtGT2 and its close orthologues, some conclusions can be made. Firstly, the gene is present in almost all analysed ascomycete plant pathogens and in several opportunistic animal pathogens (for example *Aspergillus fumigatus*). However, it is also frequently observed to be present in many saprophytic fungi including those which perform the primary events in leaf litter decay. Thus, we speculate that the importance of this gene for the evolution of fungal pathogenesis likely derived “inadvertently” from an ancient requirement for fungi to evolve the hyphal growth form, to extend filaments over solid surfaces for purposes such as nutrient acquisition. By acquiring this basic functionality, fungi also acquired a key competency which subsequently enabled them to develop parasitic (as well as potentially mutualistic) interactions with many plants and animals. Based upon all the available data it is likely that ZtGT2 synthesises or modifies a potentially widespread and essential extracellular matrix or outer cell wall polysaccharide component, which may be only a minor constituent (Fig 7). This may function to alleviate surface friction and shear stresses normally imposed on rapid hyphal tip growth over solid matrices [13, 14]. This currently structurally undefined, polysaccharide may be functionally important for many plant (and perhaps animal) pathogens and may therefore represent a viable target for future widespread control of fungal diseases. This study also emphasises the tractability of *Z*. *tritici* as a model organism for isolating genes which may be essential for contact-dependent filamentous fungal growth.

## Methods

### Plant and fungal materials

The fully genome sequenced *Z*. *tritici* isolate IPO323 was used in all experiments (http://genome.jgi-psf.org/Mycgr3/Mycgr3.home.html). For all experiments, fungal spores were initially harvested from 5 day old cultures growing (budding) on Yeast extract peptone dextrose (YPD) plates (Oxoid Ltd., Hampshire, UK) at 16°C. For RNAseq and qRT-PCR based gene expression analysis, duplicate or triplicate flasks containing 40 ml YPD broth (Oxoid Ltd., Hampshire, UK) were inoculated for 96h at 18°C (or other specified temperatures) on an orbital shaking incubator at 120 rpm. After this time, fungal materials were collected via vacuum filtration and snap frozen in liquid N_2_ until RNA extraction. Wheat seedling infection assays (cultivar Riband) was performed as described previously [48]. Briefly the adaxial surfaces of leaves were inoculated with spore suspensions collected from YPD cultures, which were washed and re-suspended in dH20 +0.01% Tween 20 to a density of 1x10^6^ spores / ml. After the indicated time, inoculated leaves were excised and snap frozen in liquid N_2_ until RNA extraction. Photographs displaying disease levels on leaves were taken twenty-one days after inoculation of the second leaf of three-week-old seedlings. All plant materials for RNAseq were prepared from duplicate (two independent) experiments performed three weeks apart. A total of six leaves were taken from six independent inoculated wheat seedlings and used to prepare a single total RNA preparation. This constituted a single biological replicate sample.

### Generation of T-DNA insertional mutants and gene deletion and complementation strains for *ZtGT2*

The T-DNA mutagenesis screen *of Z*. *tritici* was previously described [49]. TAIL PCR was carried out on fungal genomic DNA isolated from the 23–170 mutant according to previous methods [49, 50]. For targeted disruption of the *ZtGT2* gene (JGI protein model 65552; NCBI Reference Sequence: XP_003857553.1) two regions (flanks) of approximately 1000 bp of fungal genomic DNA were amplified by PCR. Flank1 was then cloned into vector pCHYG using *Sac*I and *Kpn*I (primers P1 and P2 -S5 Table) and Flank 2 using *Pst*I and *Hind*III (P3 and P4) and the resulting plasmid was transformed into *A*. *tumefaciens* strain Agl-1 via the freeze-thaw method [51]. For targeted gene deletion, a modified ΔKu70 strain of IPO323 was used [52]. To generate the complementation strain 23–170::GT2comp the entire open reading frame of *ZtGT2* plus upstream and downstream sequences was amplified by PCR on genomic DNA (primers P5 and P6). The amplicon was cloned using *Sac*I and *Kpn*I into vector pCGEN for fungal transformation of strain 23–170 and selection used 100 μg/ml Geneticin (Sigma St Louis). Agrobacterium-mediated transformation of *Z*. *tritici* was performed as previously described [24, 53]. Targeted mutants were confirmed by PCR on genomic DNA. All oligonucleotide primer sequences are provided in S5 Table.

### Identification of proteins with similarity to ZtGT2 in other fungi

The mature protein sequence of ZtGT2 (Genbank XP_003857553.1) was used for a Blastp analysis against the contents of the JGI MycoCosm fungal genome portal (http://genome.jgi.doe.gov/programs/fungi/index.jsf) in July 2017. Blastp searches at expect value homology cut-offs of 1e-100; 1e-60 and 1e-05 were performed for each taxonomic class / sub phylum. Species which returned one or more hits at each cut-off were included as a positive (harbouring a protein with the indicated level of similarity). Species returning no hits at any cut-off were deemed negative (lacking any similar proteins). Data was then presented in the form of pie charts for each taxonomic class/ sub phylum displaying number of species genomes having or lacking similar proteins at each e value cut-off, as a proportion of the total number of species genomes analysed in each category.

### Phylogenetics analysis of *ZtGT2* orthologues

Data from the protein similarity search was used for selection of sequences for phylogenetics analysis. For Maximum Likelihood gene tree analysis, the best Blastp hits were selected from 2–3 species representative of the different taxonomic subdivisions or classes from the fungal tree of life present within the JGI Mycocosm Genome portal [34]. For select species, including *Z*. *tritici* and *F*. *graminearum*, the next best Blastp hit was also included. To produce the most accurate nucleotide alignments, coding sequences were trimmed from each transcript to the conserved Type 2 glycosyltransferase domain as identified by the NCBI conserved domain database displayed in the Blastp output. The accession for the conserved domain sequence was cd06434. Genes were then aligned using MAFFT v.7.308 [54, 55], in Geneious 10.0.9 [56]. For phylogenetic reconstruction, the GTR+I+G nucleotide substitution model was selected by AIC in jModeltest 2.1.10 [57, 58]. The Maximum Likelihood phylogeny was reconstructed using PhyML [59], with the substitution model selected in jModeltest; starting tree with optimised topology, length and rate parameters; topology searching by the best of NNI and SPR; and 500 bootstraps.

### Gene deletion of the *ZtGT2* homologue (*FgGT2*) in *Fusarium graminearum*

The *FgGT2* gene, *FGRRES_00702* (http://fungi.ensembl.org/Fusarium_graminearum/Info/Index), was deleted in *F*. *graminearum* wild-type strain PH-1 (NRRL 31084) for which the complete genome sequence is available [60]. A PCR-based split-marker gene deletion strategy was chosen [35]. The DNA flanks (1000 bp 5’- and 997 bp 3’-sequence) of the *FgGT2* gene were first amplified by PCR using primers (S5 Table) pairs U650/U651 and U656/U657 and cloned into the plasmid vector pGEM-T (Promega) using the Gibson assembly Master Mix kit (New England BioLabs Inc.) according to the manufacturer’s instructions to generate vectors pMU421 and pMU422, respectively. Specific PCR was carried out in 25 μl volumes, containing 50 ng of DNA, 1 U of Expand High Fidelity *Taq-Pwo* polymerase mixture (Boehringer Mannheim), 10 pmol of each primer and 0.25 mM each deoxynucleoside triphosphate, in a standard buffer for 35 cycles with the following cycling parameters: denaturation at 94°C for 30 s; annealing at 54°C for 30 s; and DNA synthesis at 72°C for 1 min. For transformation both split-marker constructs contained in pMU421 and pMU422 were quantitatively amplified by PCR using HotStar TAQ polymerase (Qiagen) following the manufacturer’s instructions. The concentration of the PCR products was adjusted to 2 μg μl^-1^ and 5 μl of each construct was mixed and transformed into 1x10^8^ protoplasts of *F*. *graminearum* strain as previously described [61, 62]. Recovery of transformants was accomplished in liquid TB3 medium containing hygromycin (75 μg/ml) to allow selection of mutants which might otherwise be impaired in hyphal growth on selective solid agar. 0.2 ml transformation mix was added to 10 ml TB3 liquid medium containing 75 μg/ml hygromycin contained in 50 ml Falcon tubes. Cultures were grown for further 10 days in a shaking incubator set to 28 C and 180 rpm. Hygromycin resistant transformants were then transferred to PDA agar plates containing hygromycin (10 μg/ml) for further analysis. Fungal genomic DNA was extracted from transformants grown in 10 ml potato dextrose medium in the presence of hygromycin (10 μg/ml) as described [63].

In the two isolated gene replacement mutants MU426 and MU427 two diagnostic PCR fragments of 1.5kb and 1.3 kb size are detectable using oligomer pairs U659/U667 (PCR 1) and U660/U668 (PCR 2) respectively (Fig 4). In both mutants MU426 and MU427, the FgGT2 gene is absent (PCR 3 in Fig 4). Plant infection and pathogenicity tests on wheat (*Triticum aestivum*) plants of cultivar Bobwhite were grown and infected by point and whole-wheat spike spray inoculation with spore solutions as previously described [64]. Each experiment was performed in triplicate with similar results.

### Transmission electron microscopy (TEM)

Fungal cells were collected from the surfaces of YPD agar plates using a sterile loop. Samples were then high pressure frozen using a Leica Microsystems EM HPM100 and stored in liquid nitrogen before staining with 1% osmium and freeze substitution using acetone in a Leica Microsystems EM AFS. Following freeze substitution, the samples were stored at -20C for 24 hours then 4C for 24 hours before resin infiltration. Samples were infiltrated with an acetone:Spurr resin series and polymerised at 60C overnight. 70nm thin sections were cut using a Leica Microsystems UC7 microtome and collected on copper grids coated with formvar and carbon. Micrographs were collected using a JEOL 2011 transmission electron microscope at 200kV and a Gatan Ultrascan CCD camera.

### Scanning electron microscopy (SEM)

Approximately 5mm square regions were cut from samples and attached to aluminium stubs using a 50:50 mixture of graphite:TissueTek. The samples were plunge frozen in liquid nitrogen and transferred to the GATAN ALTO 2100 cryo prep system. Samples were etched and coated in a thin layer of gold. Micrographs were collected using a JEOL 6360 scanning electron microscope at 5kV.

### RNA sequencing and differential gene expression analysis

Total RNA was isolated from frozen materials using the TRIZOL procedure (Invitrogen). Library preparation and sequencing was performed at the Earlham Institute, Norwich Research Park, Norwich, UK. No pre-processing of the reads took place. Hisat2 (v2.0.4) was used to map the reads to *Zymoseptoria tritici* IPO323 (Ensembl fungi v30) or *Triticum aestivum Chinese Spring* (Ensembl plants v32) using default settings. Cuffdiff (v2.2.1) was used to produce FPKM and differential testing of counts with a classic-fpkm library normalisation method, pooled dispersion estimation method, and bias correction with effective length correction, for wheat using Ensembl plants v32 annotation but for fungi using a custom gff3 annotation file https://doi.org/10.6084/m9.figshare.4753708.v1 [42] to count against only these models. CummeRbund (v2.8.2) was used to produce the PCA plots in R (v3.1.2). Annotations were added to the cuffdiff output using Blast2Go (v3.3.5) with Blast2Go GO database 05/2016 and default filtering settings, using input from InterPro (v58.0) and Timelogic DeCypher with the NCBI NR database (23/06/16) using an e-value threshold of 1e-2. For robust differential expression analysis, only genes with fold changes of Log2 >1.5 (*Padj* 0.05) were considered further.

### Quantitative RT-PCR analysis

Total RNA was isolated from freeze-dried, fungal material collected from the stated liquid culture or from fungal infected leaf tissues, was prepared using the TRIZOL procedure (Invitrogen). Total RNA was used for all RT-PCR and Real-Time RT-PCR analyses. First-strand cDNA was synthesised from total RNA using the SuperScript III First_Strand Synthesis System for RT-PCR (Invitrogen). A 5 μg aliquot of total RNA primed with oligo(dT)_20_ was used in a 20 μl reaction, following the suppliers instructions. The resulting cDNA was analysed by Real-Time RT-PCR using a QuantiTect SYBR Green PCR Kit (Qiagen), following the supplier’s instruction. A 0.5 μl aliquot of cDNA was used in each 20 μl PCR reaction, with an annealing temperature of 60°C. Primers were used at a final concentration of 0.25 μM. Real-Time RT-PCR reactions were run for 40 cycles and analysed using an ABI 7500 Real Time PCR System. The relative expression of each fungal or plant gene was determined by normalisation with the constitutively expressed *Z*. *tritici beta-tubulin* gene [48] or with the *TaCdc48* gene for *T*. *aestivum* [31]. All oligonucleotides used are listed in S5 Table.

## Supporting information

S1 Fig The morphology and rate of yeast like growth of 23–170 is comparable to that of wild type *Z*. *tritici*. Wild type or 23–170 mutant spore suspensions at 10^4^ spores / ml in water was inoculated (5 μl) onto a YPD plate. The inoculated region was photographed at 24h intervals for a macroscopic analysis (upper panels). Middle panels display the typical characteristic spore morphologies of each strain grown on YPD agar coated slides for 24 hours. Lower panels show spores under higher magnification stained with the chitin binding fluorophore calcoflour white.
(TIF)

S2 Fig The 23–170 aberrant hyphal growth phenotype is observed at all tested water agar strengths. A spore suspension of 10^4^ spores / ml in water was inoculated (5 μl) onto a water agar plate at the indicated agar concentration. Plates were incubated at RT then photographed after 10 days.
(TIF)

S3 Fig The 23–170 aberrant hyphal growth phenotype is also observed on both Czapek-Dox and Potato dextrose agar. A spore suspension of 10^4^ spores / ml water was inoculated (5 μl) onto the surface of the indicated agar plate. Plates were incubated at RT then photographed after 21 days.
(TIF)

S4 Fig ΔZtGT2 mutants display short sinusoidal hyphae on both Czapek-Dox and Potato dextrose agar plates. A spore suspension of 10^4^ spores / ml water of wild-type or ΔZtGT2 was inoculated (5 μl) onto the surface of the indicated agar plate. Plates were incubated at RT then photographed after 21 days (Left panels). Hyphal morphology radiating from the colony edge was studied by light microscopy (right panels).
(TIF)

S5 Fig Solid media-based selection drives generation of heterokaryotic deletion mutants of the *Fusarium graminearum ZtGT2* orthologue, *FgGT2*. Gene replacement strategy for the ZtGT2 orthologue from *F*. *graminearum* with gene Id *FG00702*.*1* (http://fungi.ensembl.org/Fusarium_graminearum/Info/Index) (A) Genomic left border and right border regions (white bars) were amplified with primers and fused to parts of the *hph* hygromycin resistance gene. Fused PCR fragments were used in a split-marker strategy to replace FGRRES_00702. Horizontal black bars represent genomic areas outside the replacement construct, vertical black bars represent 3 introns. (B) Anticipated diagnostic PCR for successful gene replacement of FGRRES_0702. (C) Results of diagnostic PCR and expected sizes indicated in (A) and (B). Transformants S#11, S#19 and S#24 have the ΔFGRRES_00702 null allele but also retain a wild-type gene (heterokaryons). This was observed for all hygromycin resistant strains originating from 4 independent transformations. M—λ DNA-BstEII digest; WT—wild type. (D) Growth of *F*. *graminearum* heterokaryotic strains carrying both a WT and FGRRES_00702 null allele on potato dextrose agar after 72 hours incubation. No alteration in hyphal growth was observed. Top left to bottom right: WT, FG transformants S#10, 11, 15, 19, 24.
(TIF)

S6 Fig ΔFgGT2 mutants are still competent in invasive growth. Penetration of cellophane membranes by *F*. *graminearum* strains. Fungal spores were plated and grown for 2 d at 22°C on top of cellophane membrane on the surface of PDA plates (images labelled “Before”). The cellophane with the fungal colonies was removed and plates were incubated for an additional two days to determine whether fungal growth occurred on the plates, indicating penetration of the cellophane disk (images labelled “After”). The *ΔFgMAP1* mutant strain was used as a control strain shown previously to be defective in cellophane and plant penetration [64].
(TIF)

S7 Fig Anti-ZtGT2 peptide antibodies recognise a specific protein present in the cell wall fraction of *Z*. *tritici*. Fungal mycelium grown at 25°C in liquid YPD was subjected to crude protein fractionation and western blot analysis with an anti-ZtGT2 peptide antibody. (A) The antiserum detects a ~56kDa protein found only in the cell wall fraction of wild type fungal cells (B) The specific binding to this protein in wild type cells is confirmed by a peptide competition experiment.
(TIF)

S8 Fig Additional TEM images of the varied surface structural abnormalities observed in ΔZtGT2 mutants. Arrows highlight the different surface structures observed in the mutants.
(TIF)

S9 Fig Monosaccharide composition analysis of alcohol insoluble residue (AIR) from cell walls and culture filtrates of ΔZtGT2 and wild-type strains. Fungal strains were grown in shaking culture flasks until saturation and then separated by filtration. AIR was generated from cell walls, samples were hydrolysed and monosaccharaides levels (glucose, mannose and galactose) were quantified (A). Culture filtrates were ethanol precipitated and then analysed the same way (B). Data shown derives from three biological replicates and in each case and monosaccharide levels are expressed in milligrams / gram of total dry weight (mg /g. dwt).
(TIF)

S10 Fig The ΔZtGT2 strains are not affected in ability to adhere to hydrophobic plastic or leaf surfaces. Conidial suspensions were left to adhere to either plastic petri dishes (upper panels) before extensive washing and counting of remaining adhered spores (from micrograph images taken before and after washing). Lower panels- cell suspensions were applied to surface of wheat leaves or to glass coverslips and allowed to adhere. Leaves or coverslips were then washed by vortexing and the resulting suspensions plated out on YPD agar + 100 μg/ml G418 antibiotic. Plates were incubated for 6 days and colonies were counted from photographic images. A decrease in the number of colony forming units (cfus) retrieved from leaf surfaces relative to glass coverslips was taken as indicative of adhesion. There were no statistical differences between wild-type and ΔZtGT2 strains from all assays.
(TIF)

S11 Fig ΔZtGT2 mutants exhibit no change in susceptibility to chemical, oxidative, osmotic or temperature stress. Wild type; 23–170 mutant; ΔZtGT2 and 23–170::GT2comp spore suspensions of initially 10^6^ spores / ml (or three successive 3-fold dilutions in water), were inoculated (5 μl) onto a YPD agar plates containing the added stress agent. Plates were then photographed after growth at 16°C or 30°C for six days.
(TIF)

S12 Fig Diagrammatic summary of the workflow for RNAseq sample generation and subsequent analysis. (TIF)

S13 Fig A family of fungal transmembrane CFEM domain containing proteins have reduced expressed in the ΔZtGT2 mutants. Seven genes in this category show significantly reduced expression in ΔZtGT2 mutants growing both in liquid culture and on leaf surfaces.
(TIF)

S14 Fig ΔZtGT2 mutants over-express several candidate secreted proteins during growth in liquid culture including the *Zt3LysM* chitin binding virulence effector. (TIF)

S15 Fig *Zt3LysM* overexpression in ΔZtGT2 is independent of the culture broth used. An independent experiment was performed growing the wild type and ΔZtGT2 fungus in either YPD or PDB broth for 5 days. Real-Time qRT-PCR was then used to measure relative expression of the *Zt3LysM* effector. Data was normalised to the expression of the *Z*. *tritici beta tubulin* gene and presented as fold change relative to gene expression by the WT fungus in YPD broth.
(TIF)

S16 Fig Independent qPCR validation of wheat defence-associated genes activated during early infection by wild type and ΔZtGT2 strains analysed at 24 and 48 hpi. (A) indicates the number of transcripts with higher expression in wheat leaves inoculated with WT fungus than with ΔZtGT2. (B) qRT-PCR validation of expression of the two indicated wheat *PR* genes. Data is normalised to expression of the wheat *cdc48* gene.
(TIF)

S1 Table Blastp analysis on fungal genomes for proteins with similarity to ZtGT2. (XLSX)

S2 Table Fungal species list used for phylogenetic analysis. (DOCX)

S3 Table RNAseq data for all differentially expressed *Z*. *tritici* genes. (XLSX)

S4 Table RNAseq data for all differentially expressed wheat (*Triticum aestivum*) genes. (XLSX)

S5 Table Oligonucleotides used in this study. (XLSX)

S1 Text Supplementary methods. (DOCX)
