# Supplementary figures and images for: A conserved fungal glycosyltransferase facilitates pathogenesis of plants by enabling hyphal growth on solid surfaces
Source: PLoS Pathog. 2017 Oct 11;13(10):e1006672. doi: 10.1371/journal.ppat.1006672 (PMC5653360; doi:10.1371/journal.ppat.1006672)

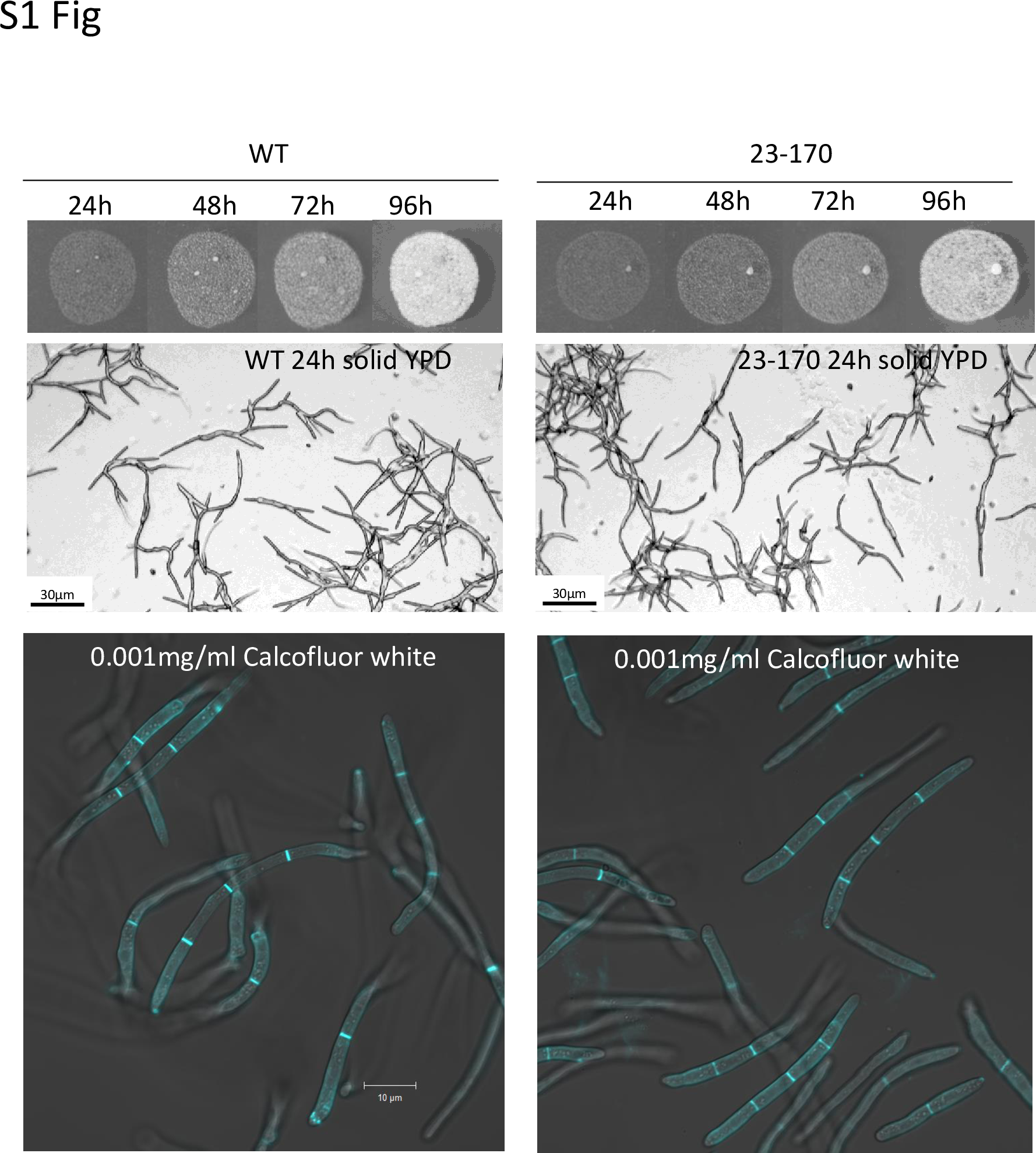

Supplement: S1 Fig — Wild type or 23–170 mutant spore suspensions at 104 spores / ml in water was inoculated (5 μl) onto a YPD plate. The inoculated region was photographed at 24h intervals for a macroscopic analysis (upper panels). Middle panels display the typical characteristic spore morphologies of each strain grown on YPD agar coated slides for 24 hours. Lower panels show spores under higher magnification stained with the chitin binding fluorophore calcoflour white. (TIF) [file ppat.1006672.s001.tif]

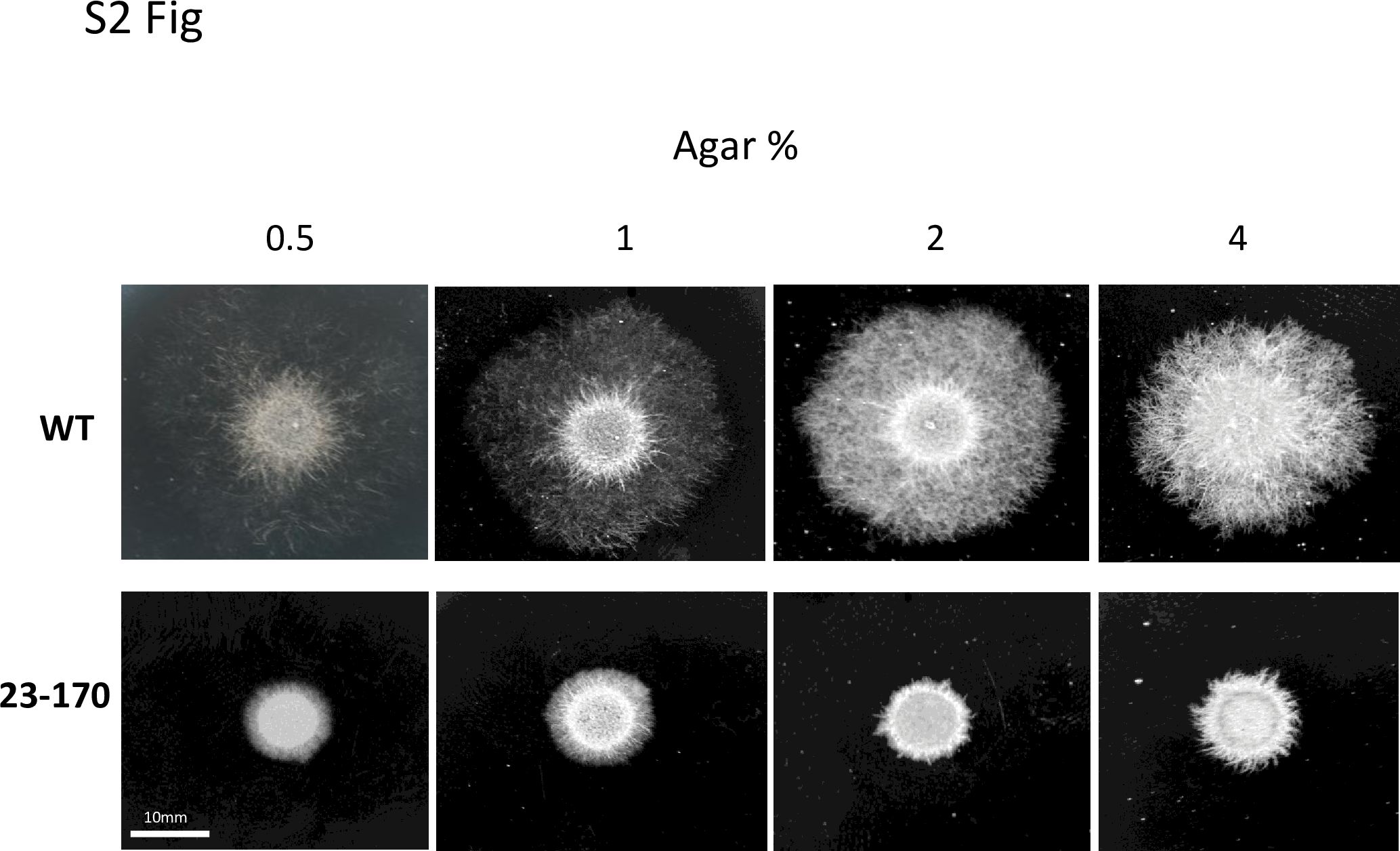

Supplement: S2 Fig — A spore suspension of 104 spores / ml in water was inoculated (5 μl) onto a water agar plate at the indicated agar concentration. Plates were incubated at RT then photographed after 10 days. (TIF) [file ppat.1006672.s002.tif]

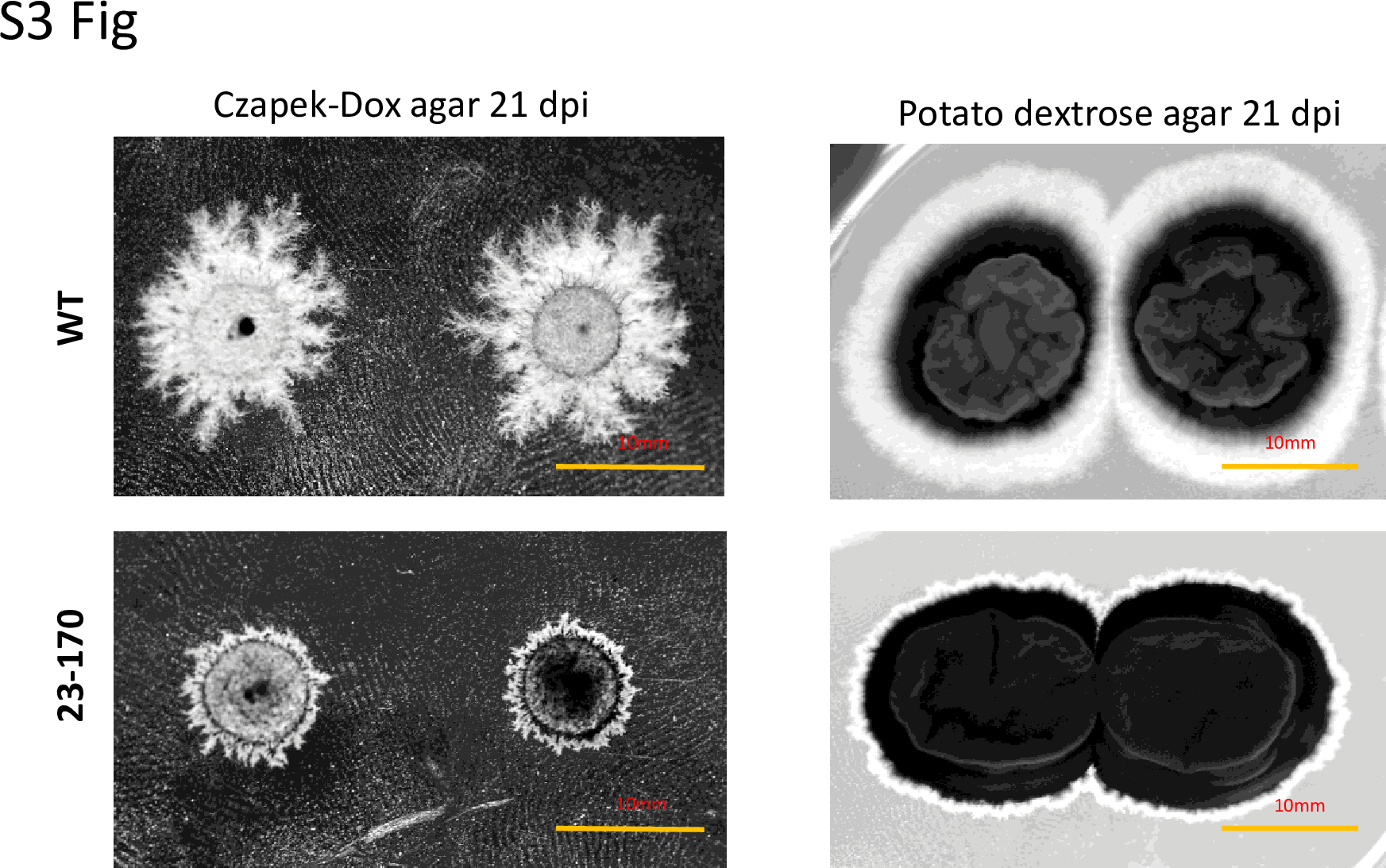

Supplement: S3 Fig — A spore suspension of 104 spores / ml water was inoculated (5 μl) onto the surface of the indicated agar plate. Plates were incubated at RT then photographed after 21 days. (TIF) [file ppat.1006672.s003.tif]

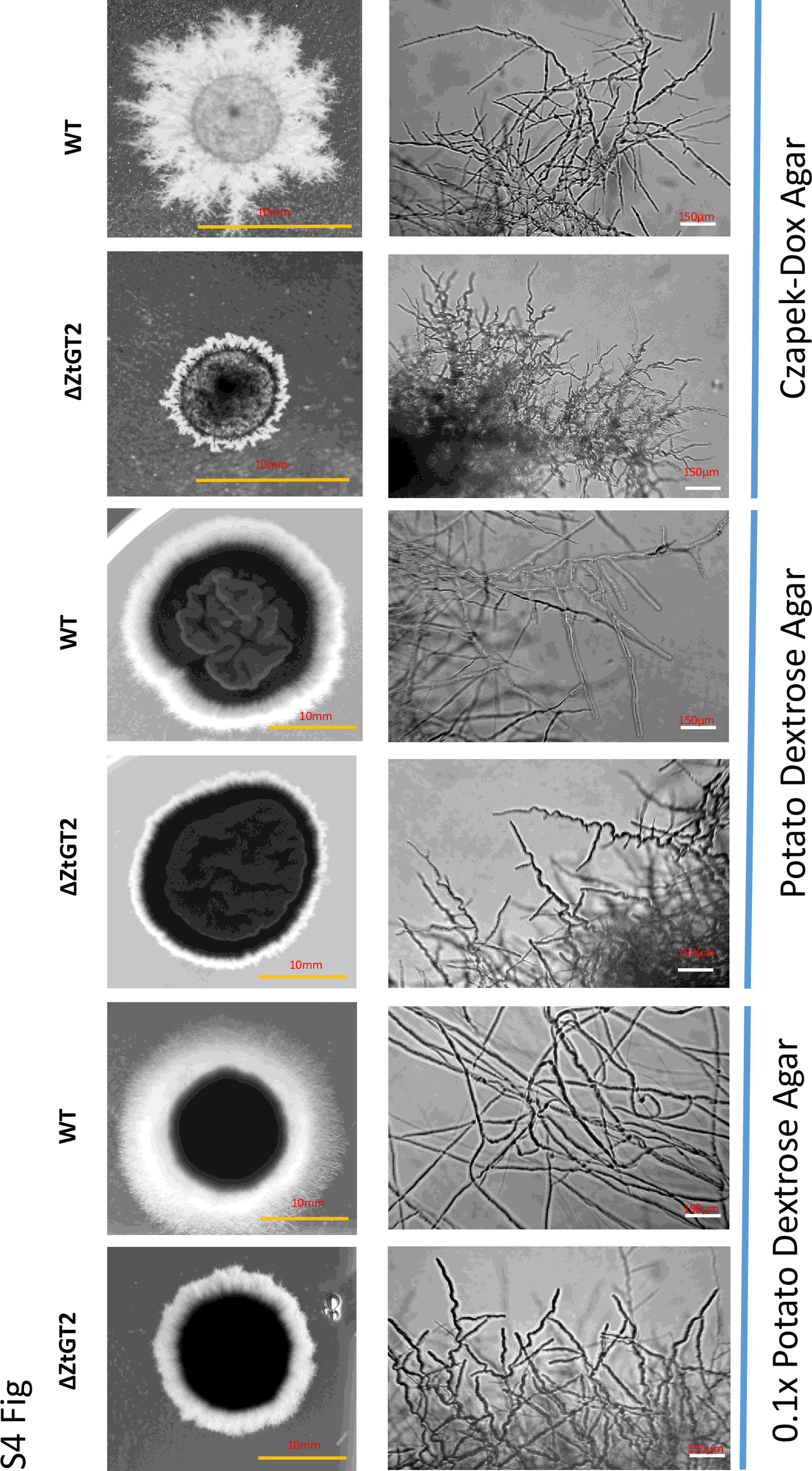

Supplement: S4 Fig — A spore suspension of 104 spores / ml water of wild-type or ΔZtGT2 was inoculated (5 μl) onto the surface of the indicated agar plate. Plates were incubated at RT then photographed after 21 days (Left panels). Hyphal morphology radiating from the colony edge was studied by light microscopy (right panels). (TIF) [file ppat.1006672.s004.tif]

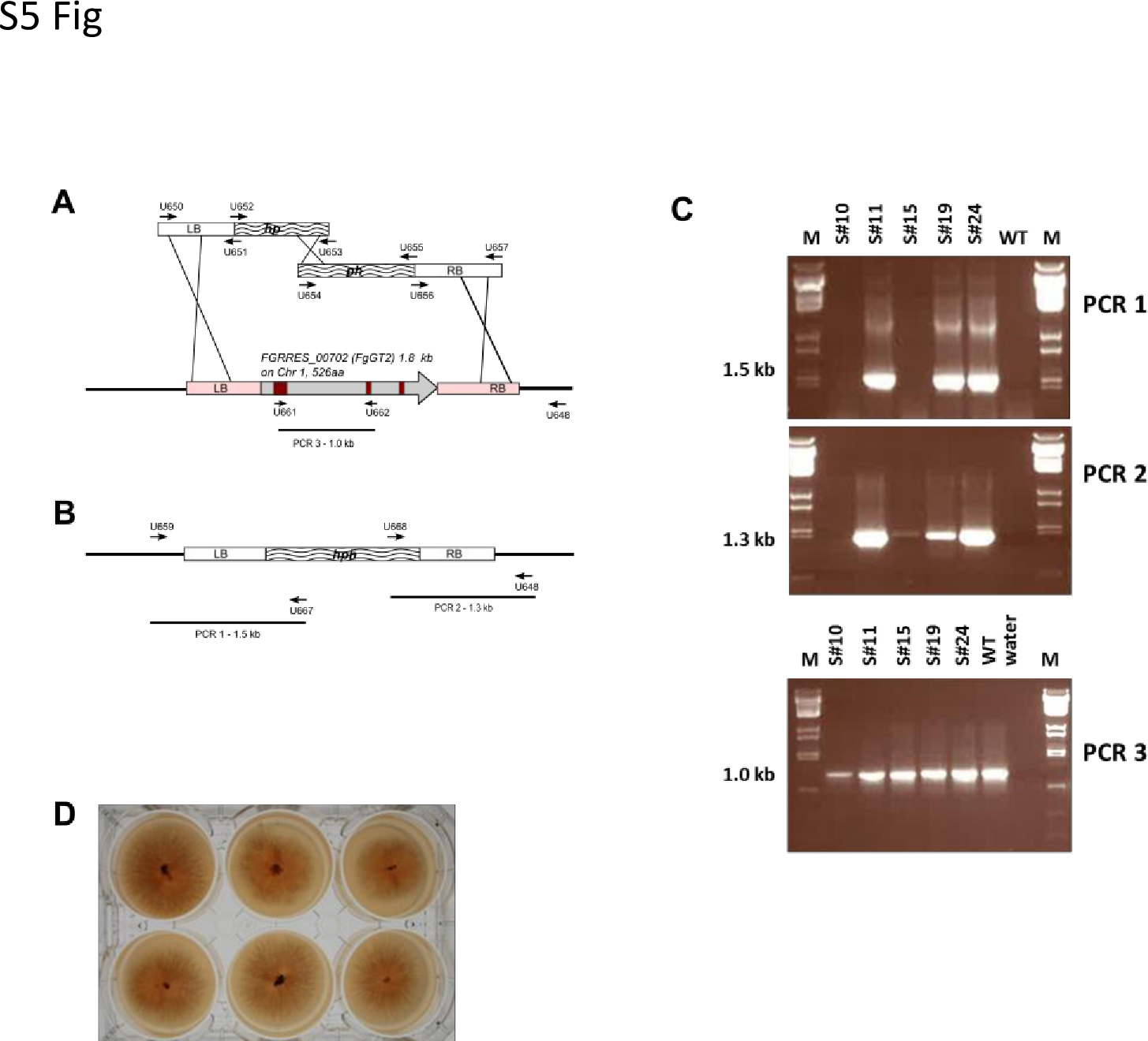

Supplement: S5 Fig — Gene replacement strategy for the ZtGT2 orthologue from F. graminearum with gene Id FG00702.1 (http://fungi.ensembl.org/Fusarium_graminearum/Info/Index) (A) Genomic left border and right border regions (white bars) were amplified with primers and fused to parts of the hph hygromycin resistance gene. Fused PCR fragments were used in a split-marker strategy to replace FGRRES_00702. Horizontal black bars represent genomic areas outside the replacement construct, vertical black bars represent 3 introns. (B) Anticipated diagnostic PCR for successful gene replacement of FGRRES_0702. (C) Results of diagnostic PCR and expected sizes indicated in (A) and (B). Transformants S#11, S#19 and S#24 have the ΔFGRRES_00702 null allele but also retain a wild-type gene (heterokaryons). This was observed for all hygromycin resistant strains originating from 4 independent transformations. M—λ DNA-BstEII digest; WT—wild type. (D) Growth of F. graminearum heterokaryotic strains carrying both a WT and FGRRES_00702 null allele on potato dextrose agar after 72 hours incubation. No alteration in hyphal growth was observed. Top left to bottom right: WT, FG transformants S#10, 11, 15, 19, 24. (TIF) [file ppat.1006672.s005.tif]

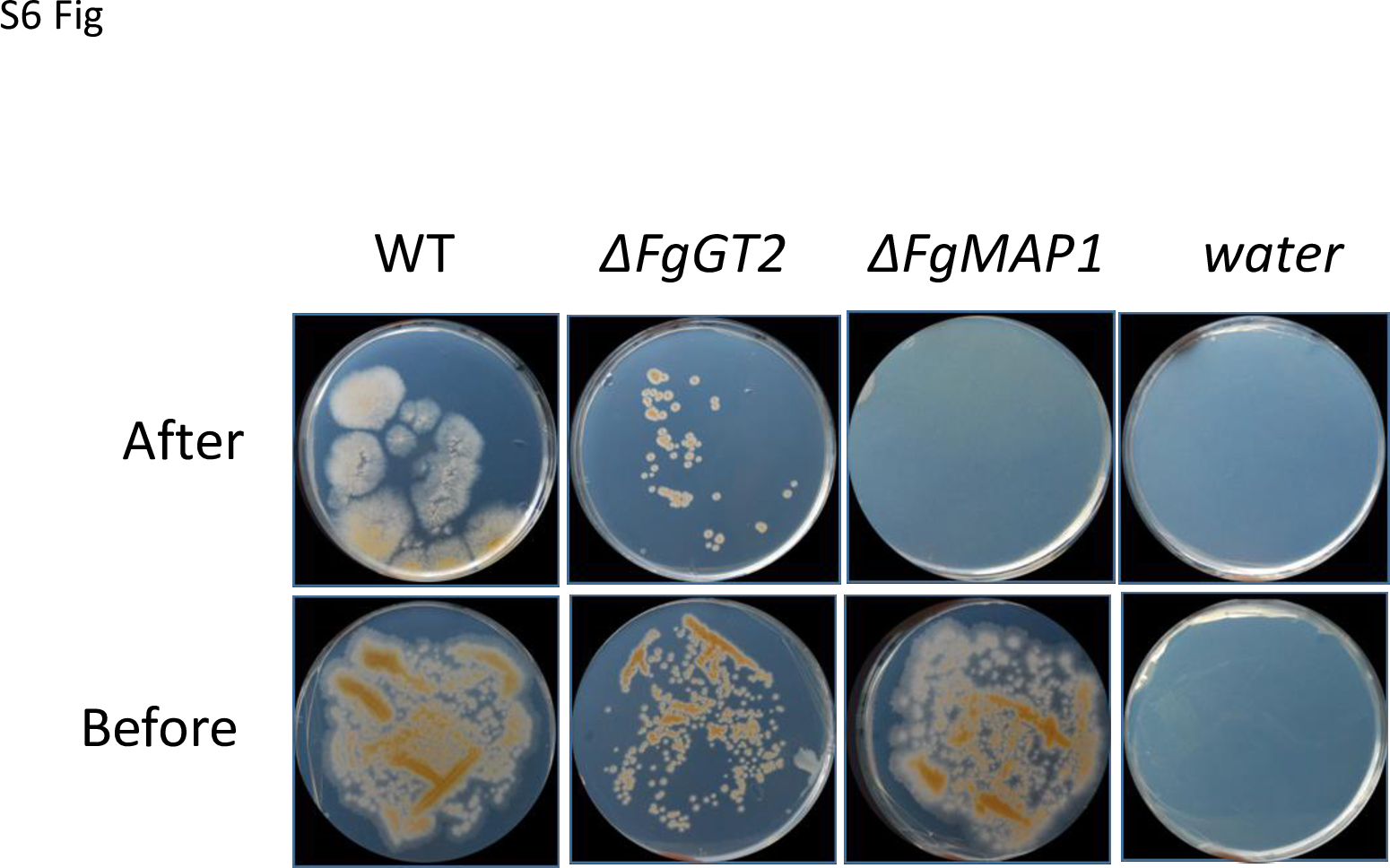

Supplement: S6 Fig — Penetration of cellophane membranes by F. graminearum strains. Fungal spores were plated and grown for 2 d at 22°C on top of cellophane membrane on the surface of PDA plates (images labelled “Before”). The cellophane with the fungal colonies was removed and plates were incubated for an additional two days to determine whether fungal growth occurred on the plates, indicating penetration of the cellophane disk (images labelled “After”). The ΔFgMAP1 mutant strain was used as a control strain shown previously to be defective in cellophane and plant penetration [64]. (TIF) [file ppat.1006672.s006.tif]

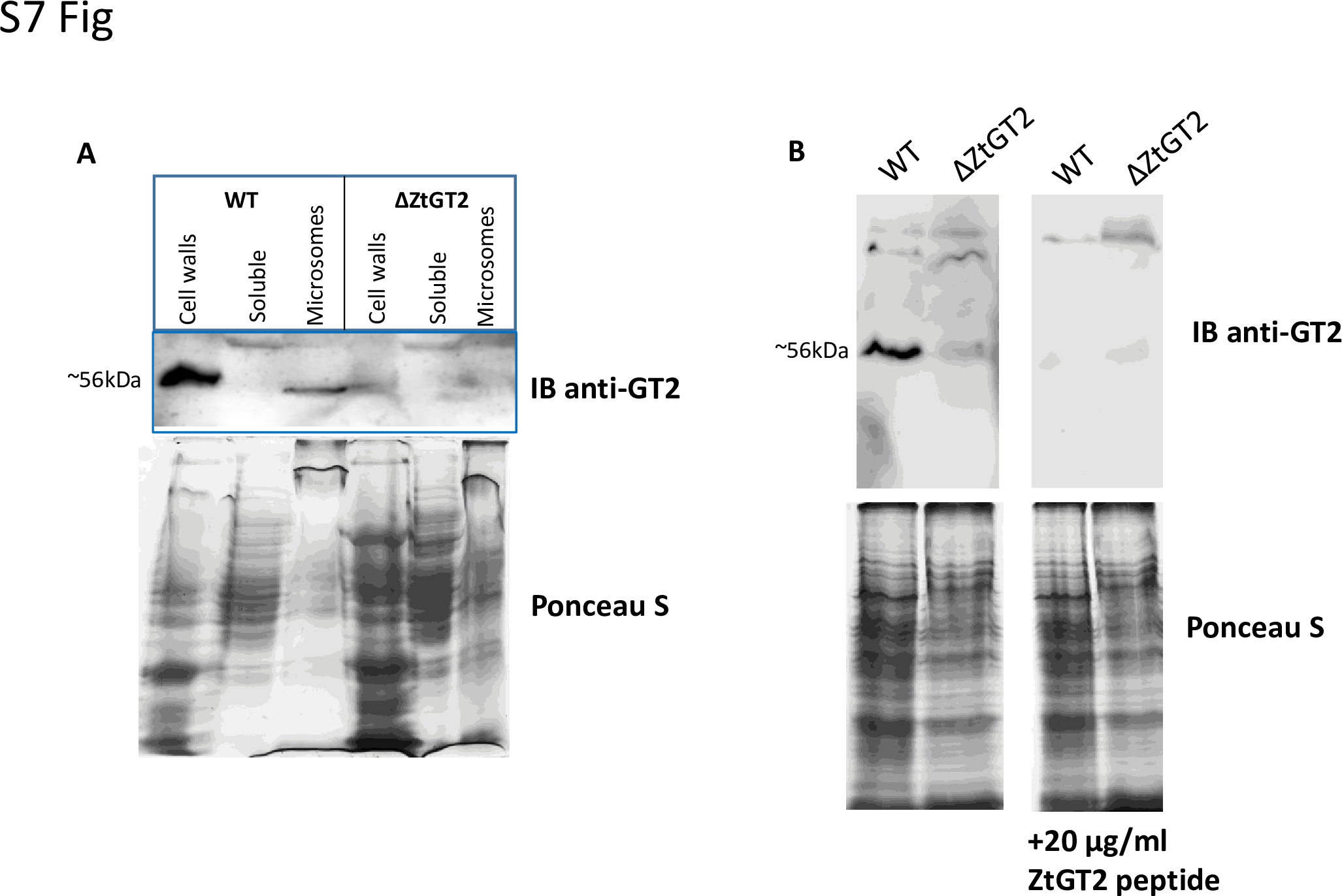

Supplement: S7 Fig — Fungal mycelium grown at 25°C in liquid YPD was subjected to crude protein fractionation and western blot analysis with an anti-ZtGT2 peptide antibody. (A) The antiserum detects a ~56kDa protein found only in the cell wall fraction of wild type fungal cells (B) The specific binding to this protein in wild type cells is confirmed by a peptide competition experiment. (TIF) [file ppat.1006672.s007.tif]

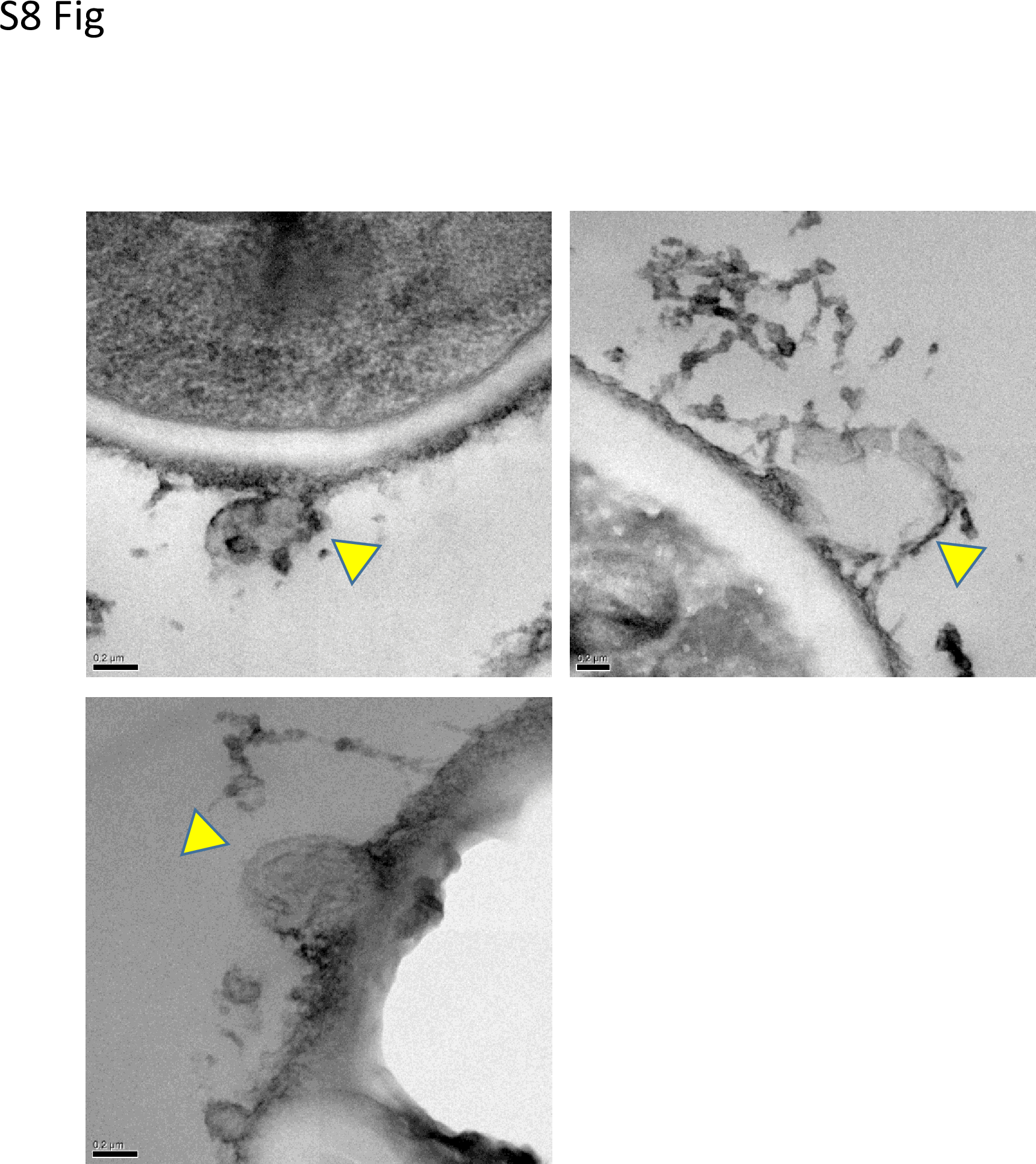

Supplement: S8 Fig — Arrows highlight the different surface structures observed in the mutants. (TIF) [file ppat.1006672.s008.tif]

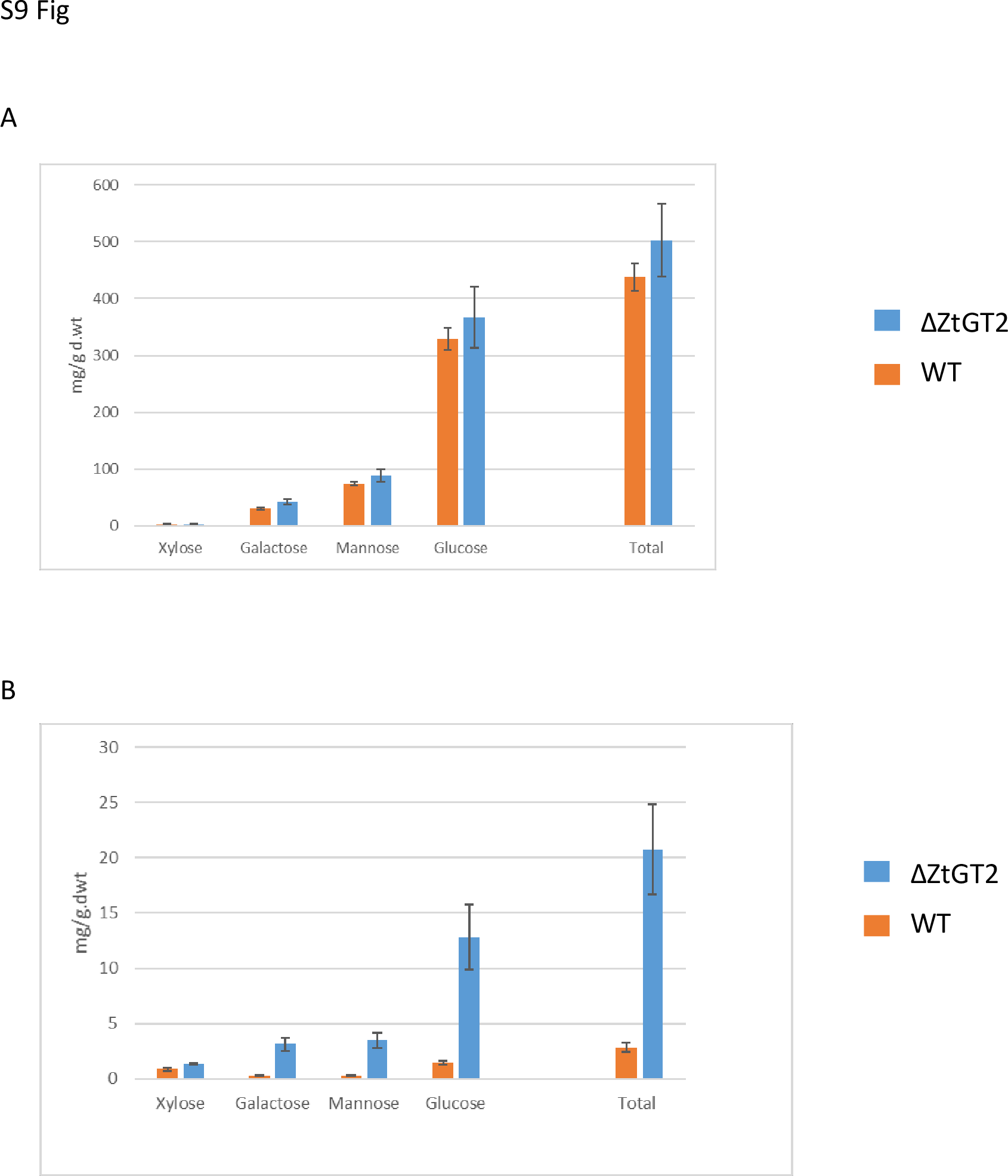

Supplement: S9 Fig — Fungal strains were grown in shaking culture flasks until saturation and then separated by filtration. AIR was generated from cell walls, samples were hydrolysed and monosaccharaides levels (glucose, mannose and galactose) were quantified (A). Culture filtrates were ethanol precipitated and then analysed the same way (B). Data shown derives from three biological replicates and in each case and monosaccharide levels are expressed in milligrams / gram of total dry weight (mg /g. dwt). (TIF) [file ppat.1006672.s009.tif]

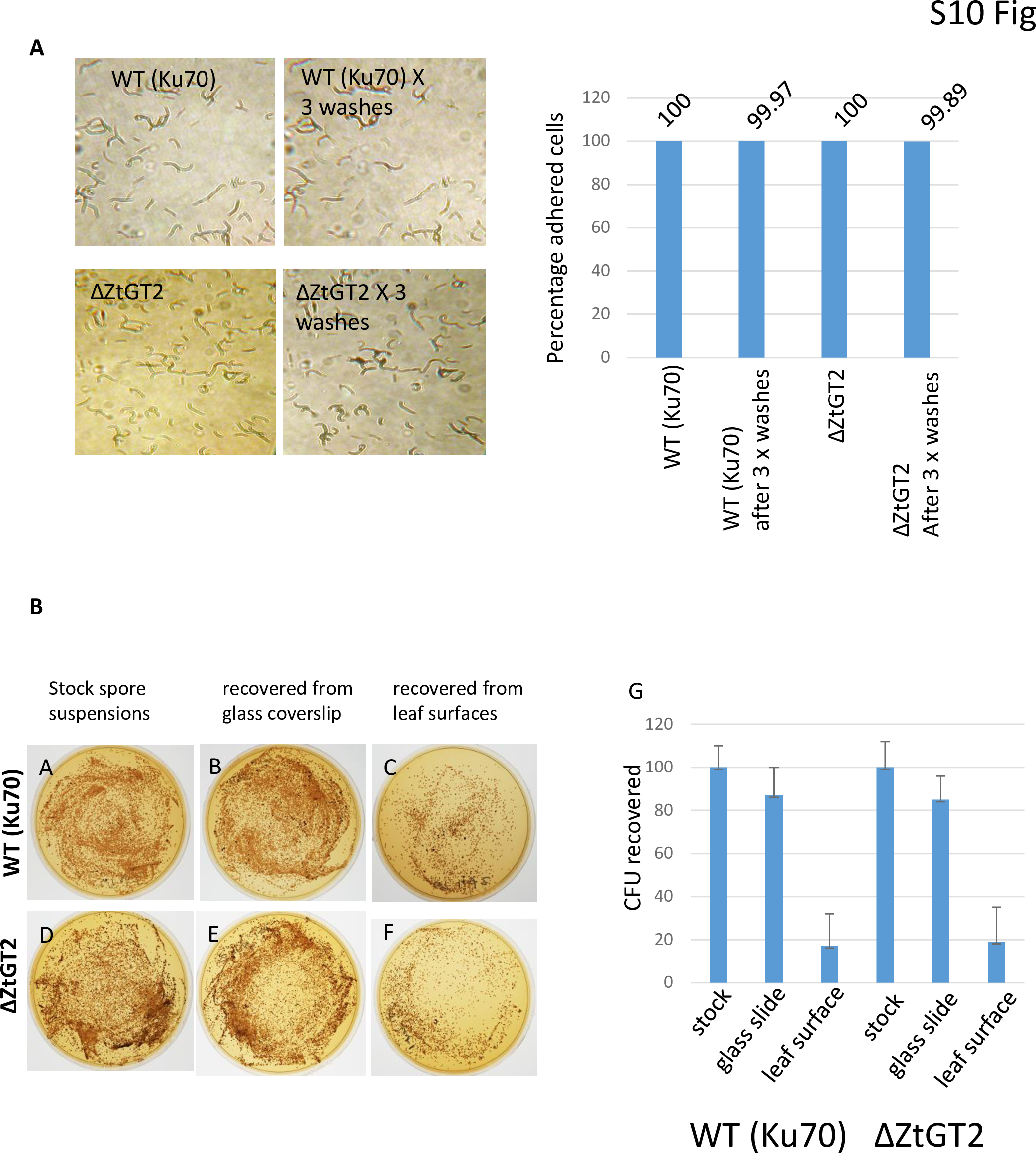

Supplement: S10 Fig — Conidial suspensions were left to adhere to either plastic petri dishes (upper panels) before extensive washing and counting of remaining adhered spores (from micrograph images taken before and after washing). Lower panels- cell suspensions were applied to surface of wheat leaves or to glass coverslips and allowed to adhere. Leaves or coverslips were then washed by vortexing and the resulting suspensions plated out on YPD agar + 100 μg/ml G418 antibiotic. Plates were incubated for 6 days and colonies were counted from photographic images. A decrease in the number of colony forming units (cfus) retrieved from leaf surfaces relative to glass coverslips was taken as indicative of adhesion. There were no statistical differences between wild-type and ΔZtGT2 strains from all assays. (TIF) [file ppat.1006672.s010.tif]

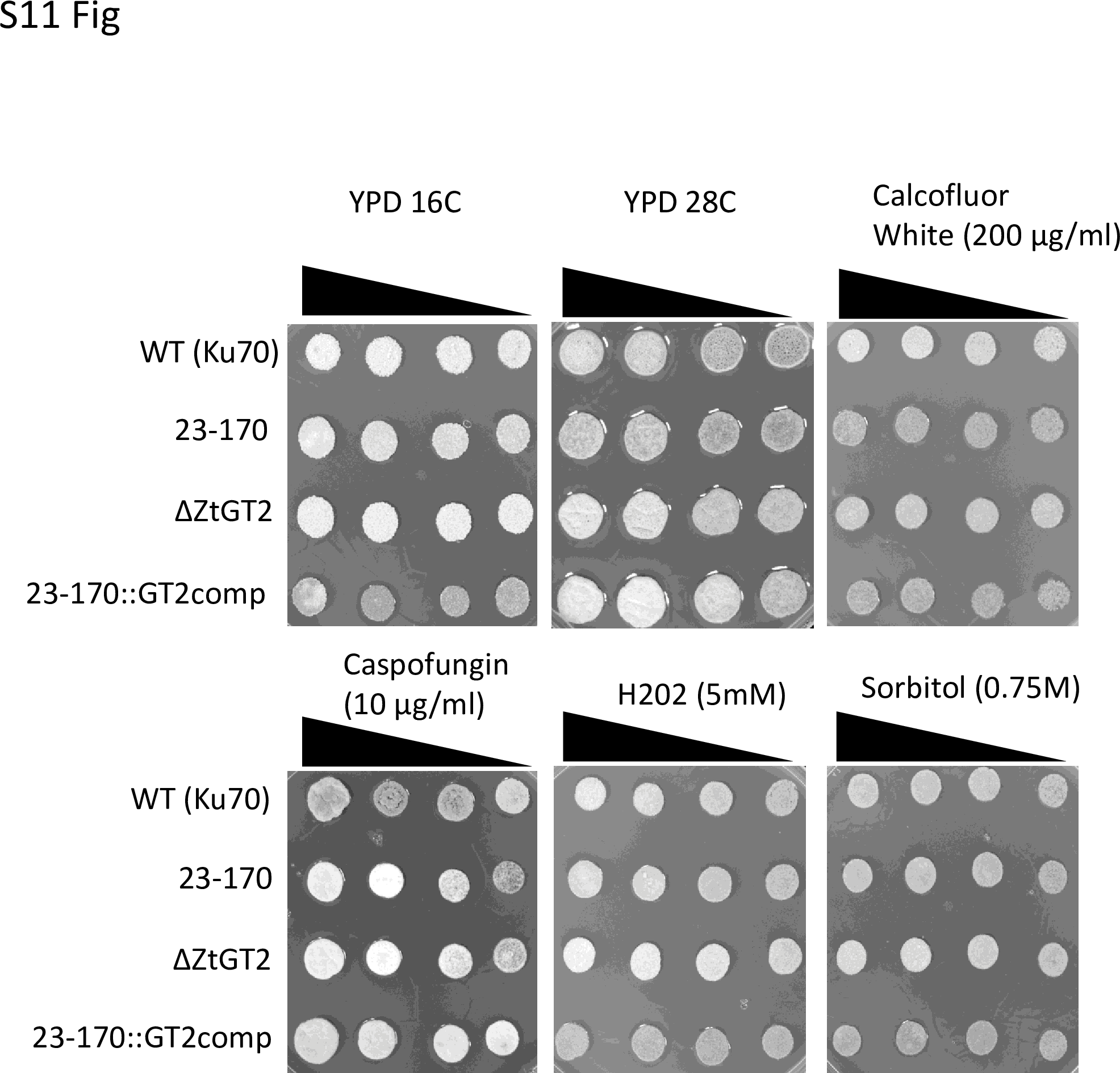

Supplement: S11 Fig — Wild type; 23–170 mutant; ΔZtGT2 and 23–170::GT2comp spore suspensions of initially 106 spores / ml (or three successive 3-fold dilutions in water), were inoculated (5 μl) onto a YPD agar plates containing the added stress agent. Plates were then photographed after growth at 16°C or 30°C for six days. (TIF) [file ppat.1006672.s011.tif]

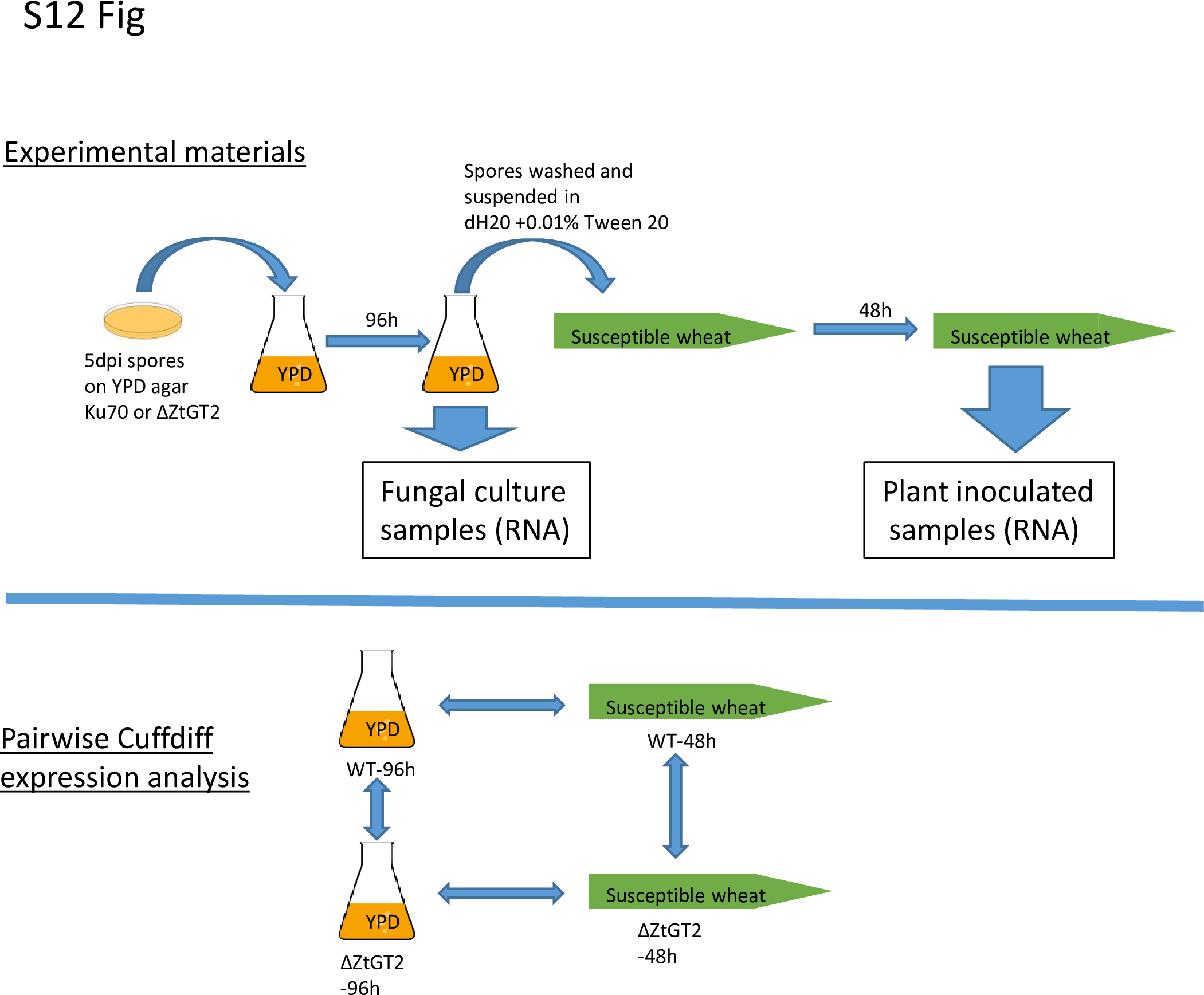

Supplement: S12 Fig — (TIF) [file ppat.1006672.s012.tif]

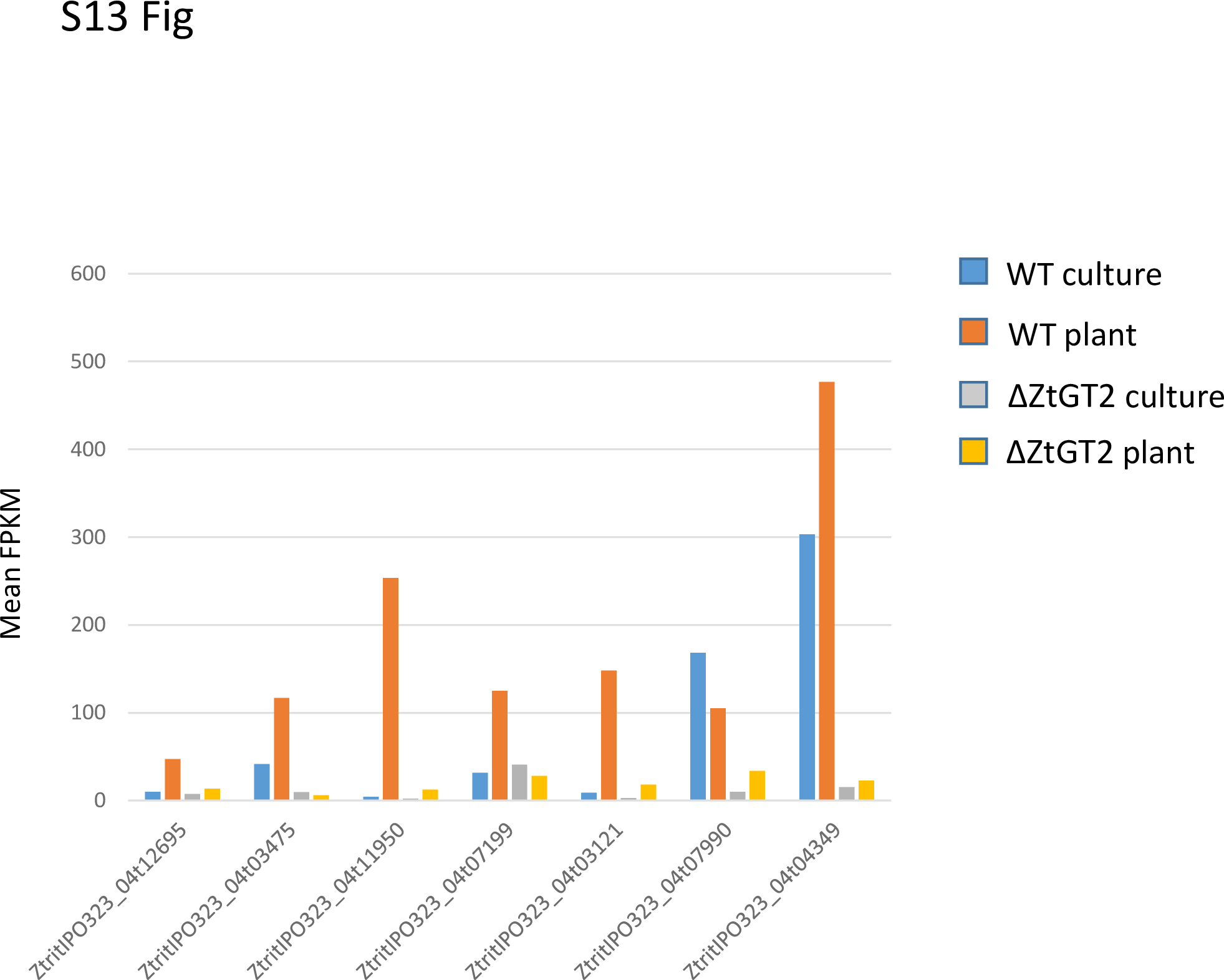

Supplement: S13 Fig — Seven genes in this category show significantly reduced expression in ΔZtGT2 mutants growing both in liquid culture and on leaf surfaces. (TIF) [file ppat.1006672.s013.tif]

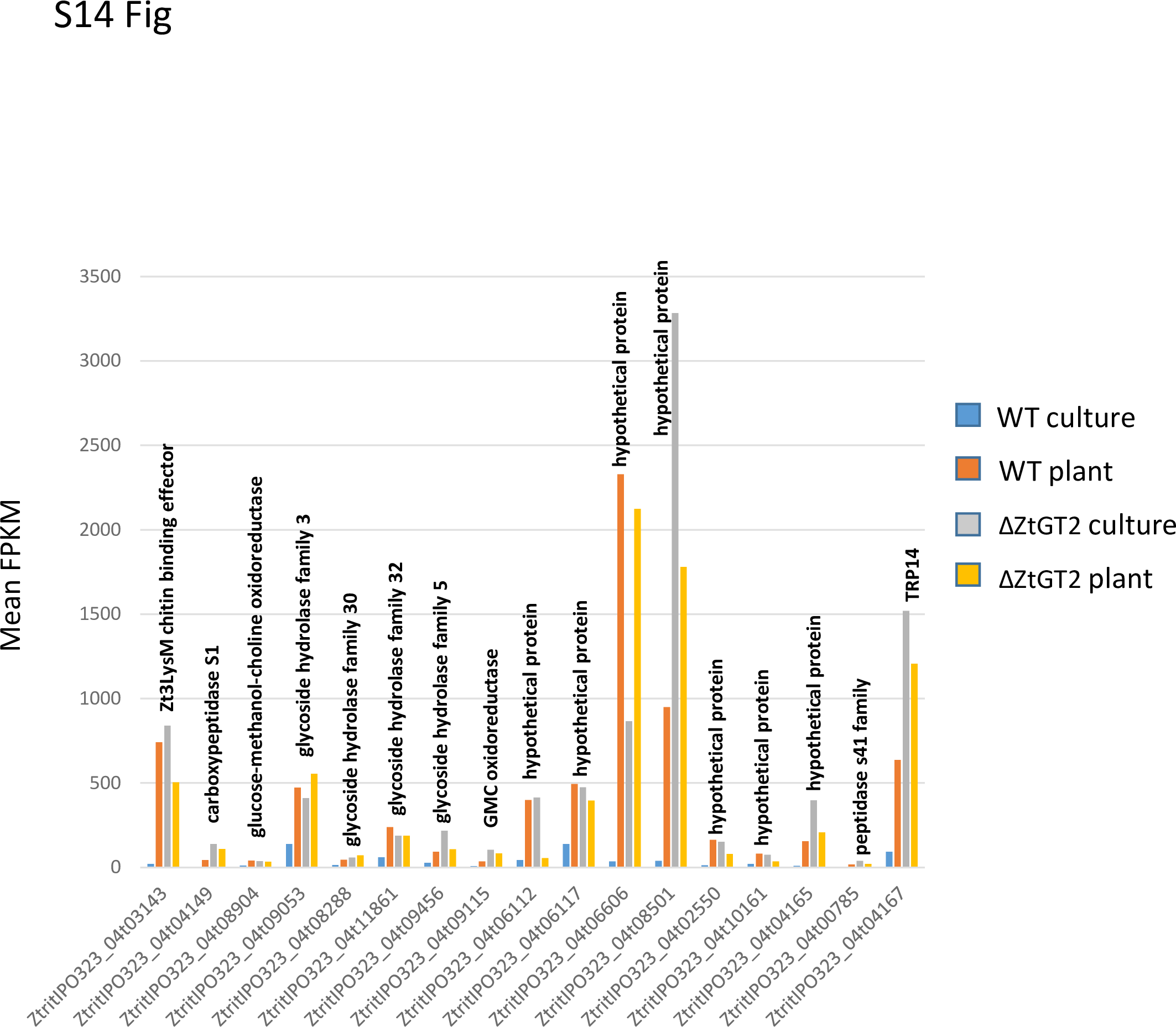

Supplement: S14 Fig — (TIF) [file ppat.1006672.s014.tif]

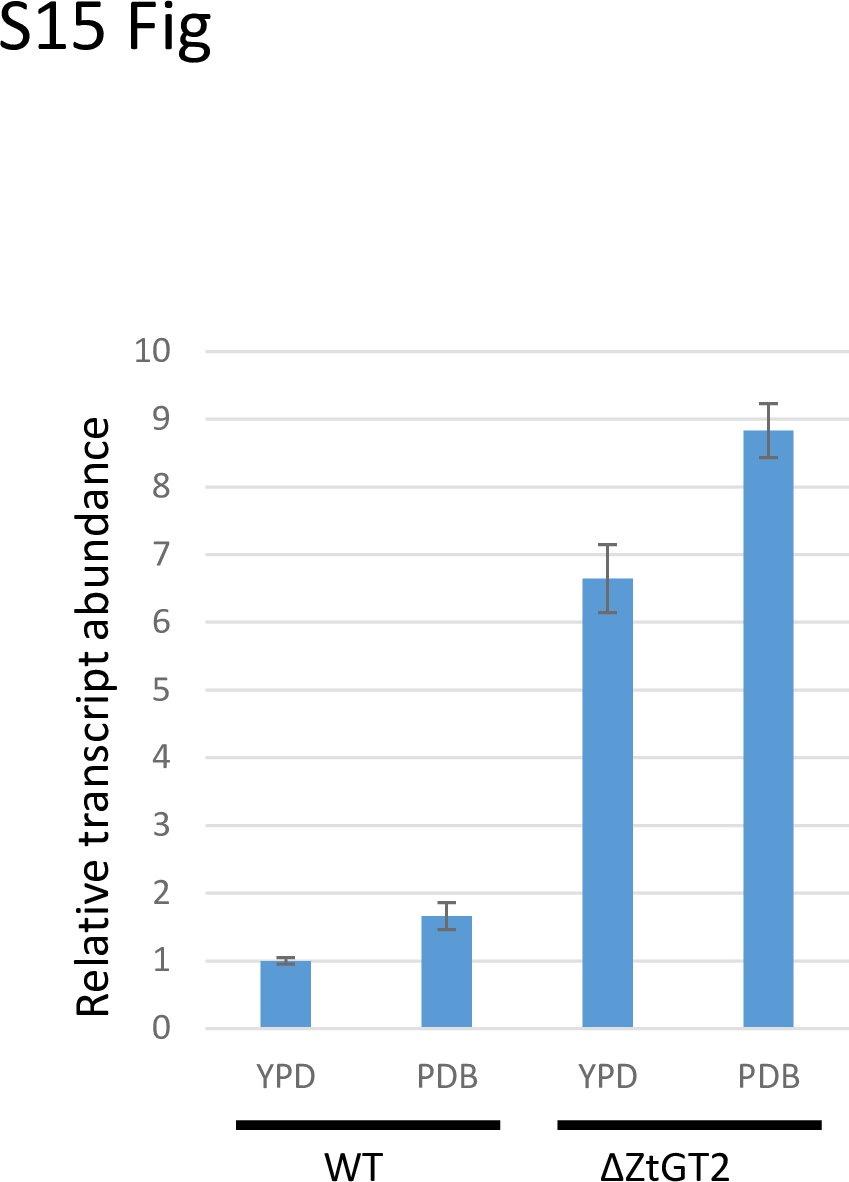

Supplement: S15 Fig — An independent experiment was performed growing the wild type and ΔZtGT2 fungus in either YPD or PDB broth for 5 days. Real-Time qRT-PCR was then used to measure relative expression of the Zt3LysM effector. Data was normalised to the expression of the Z. tritici beta tubulin gene and presented as fold change relative to gene expression by the WT fungus in YPD broth. (TIF) [file ppat.1006672.s015.tif]

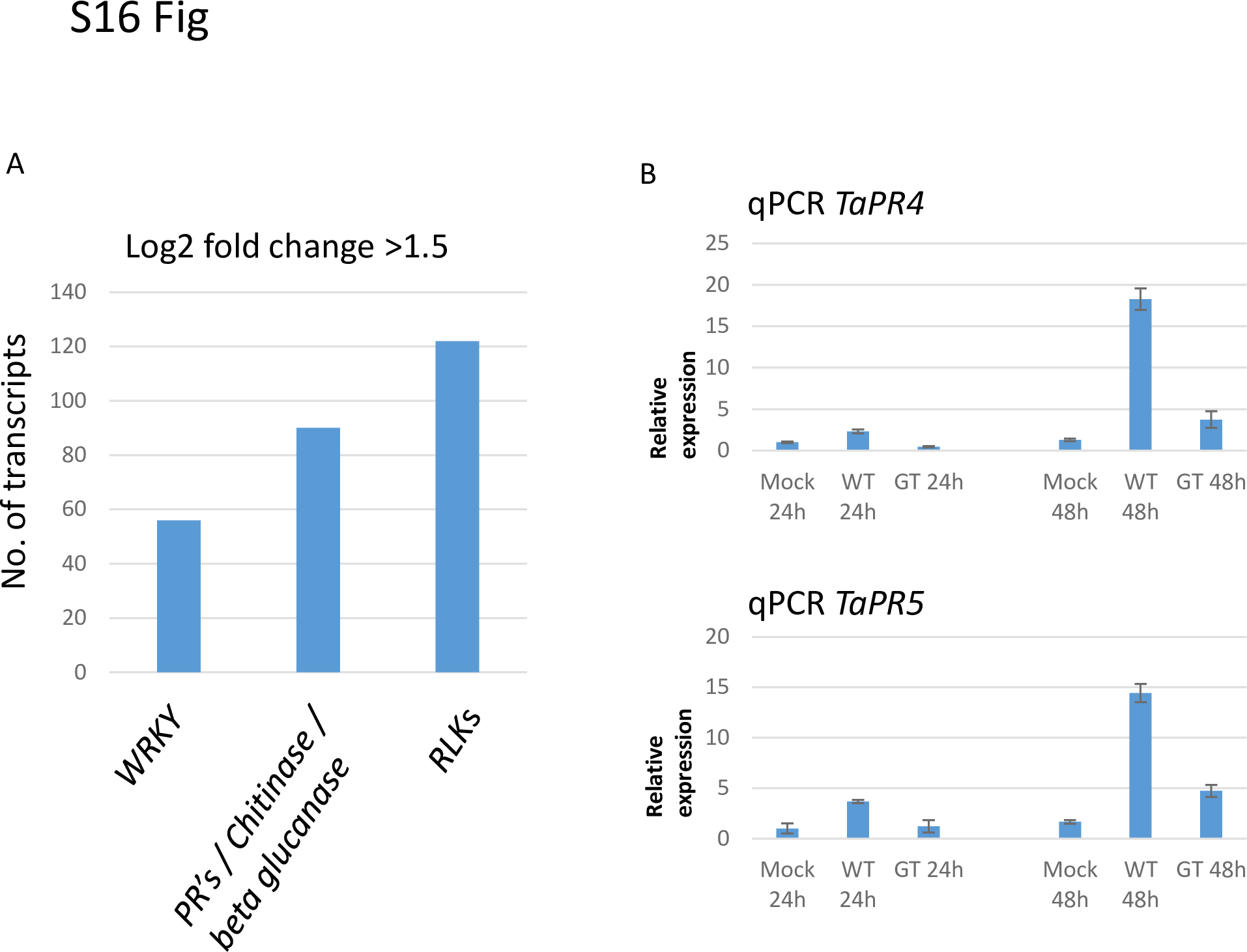

Supplement: S16 Fig — (A) indicates the number of transcripts with higher expression in wheat leaves inoculated with WT fungus than with ΔZtGT2. (B) qRT-PCR validation of expression of the two indicated wheat PR genes. Data is normalised to expression of the wheat cdc48 gene. (TIF) [file ppat.1006672.s016.tif]
