# Supplementary material for: A conserved fungal glycosyltransferase facilitates pathogenesis of plants by enabling hyphal growth on solid surfaces
Source: PLoS Pathog. 2017 Oct 11;13(10):e1006672. doi: 10.1371/journal.ppat.1006672 (PMC5653360; doi:10.1371/journal.ppat.1006672)
Supplement: S2 Table — (DOCX) [file ppat.1006672.s018.docx]

S2 Table Fungal Species used in Phylogenetic gene tree analysis

| **Species name** | **Phylum** | **Class** | **Order** | **Lifestyle description** |
| --- | --- | --- | --- | --- |
| *Zymoseptoria tritici* | Ascomycota | Dothideomycete | Capnodiales | Plant pathogen |
| *Cladosporium fulvum* | Ascomycota | Dothideomycete | Capnodiales | Plant pathogen |
| *Aureobasidium pullulans var melanogenum* | Ascomycota | Dothideomycete | Dothideales | Plant epiphyte / endophyte/ opportunistic animal pathogen |
| *Stagonospora nodorum* | Ascomycota | Dothideomycete | Pleosporales | Plant pathogen |
| *Aspergillus nidulans* | Ascomycota | Eurotiomycetes | Eurotiales | saprotroph |
| *Aspergillus fumigatus* | Ascomycota | Eurotiomycetes | Eurotiales | opportunistic animal pathogen |
| *Blastomyces dermatitidis* | Ascomycota | Eurotiomycetes | Onygenales | opportunistic animal pathogen |
| *Cladonia grayi* | Ascomycota | Lecanoromycetes | Lecanorales | Symbiotic Lichen forming fungus |
| *Xanthoria parietina* | Ascomycota | Lecanoromycetes | Teloschistales | Symbiotic Lichen forming fungus |
| *Blumeria graminis* | Ascomycota | Leotiomycetes | Erysiphales | Plant pathogen |
| *Botrytis cinerea* | Ascomycota | Leotiomycetes | Helotiales | Plant pathogen |
| *Thelebolus microsporus* | Ascomycota | Leotiomycetes | Thelebolales | Saprotroph/ opportunistic animal pathogen |
| *Fusarium graminearum* | Ascomycota | Sordariomycetes | Hypocreales | Plant pathogen |
| *Cordyceps militaris* | Ascomycota | Sordariomycetes | Hypocreales | Saprotroph/ insect (animal) pathogen |
| *Trichoderma harzianum* | Ascomycota | Sordariomycetes | Hypocreales | Saprotroph/fungal anatagonist |
| *Symbiotaphrina kochii* | Ascomycota | Xylonomycetes | Symbiotaphinales | Insect symbiont |
| *Trinosporium guianense* | Ascomycota | Xylonomycetes | Xylonomycetales | Fungal symbiont |
| *Arthrobotrys oligospora* | Ascomycota | Orbiliomycetes | Orbiliales | Saprotroph/ nematode (animal) pathogen |
| *Monacrosporium haptotylum* | Ascomycota | Orbiliomycetes | Orbiliales | Saprotroph/ nematode pathogen |
| *Cryptococcus neoformans var neoformans* | Basidiomycota | Tremellomycetes | Tremellales | Saprotroph/ opportunistic animal pathogen |
| *Rhizopus microsporus* | Mucoromycota | Mucoromycetes | Mucorales | Plant pathogen |
| *Batrachochytrium dendrobatidis* | Chytridiomycota | Chytridiomycetes | Rhizophydiales | Amphibian (animal) pathogen |
