# Supplementary material for: A conserved fungal glycosyltransferase facilitates pathogenesis of plants by enabling hyphal growth on solid surfaces
Source: PLoS Pathog. 2017 Oct 11;13(10):e1006672. doi: 10.1371/journal.ppat.1006672 (PMC5653360; doi:10.1371/journal.ppat.1006672)
Supplement: S1 Text — (DOCX) [file ppat.1006672.s022.docx]

S1 Supplementary methods text for-

**A conserved fungal Glycosyltransferase facilitates pathogenesis of plants by enabling hyphal growth on solid surfaces.**

Robert King^1¶^, Martin Urban^2¶^, Rebecca P Lauder^3¶^, Nichola Hawkins^4^, Matthew Evans^5,6^, Amy Plummer^5^, Kirstie Halsey^3^, Alison Lovegrove^5^, Kim Hammond-Kosack^2^, Jason J Rudd^2*^.

**Supplementary methods sections**

**Plant and fungal materials**

For analysis of the ability of strains to form hyphae *in vitro*, spores collected from YPD plates were streaked onto sterile tap water agar plates (1% agar) and visualized after 10 days. For analysing the sensitivity of spores to calcofluor white, caspofungin, sorbitol or H_2_O_2_ (all Sigma St Louis), sterile filtered stock solutions were dissolved in YEPD agar. Serial dilutions of spores suspended in sterile water were then applied as 5 μl droplets. Photographs of the relative colony densities were taken after 6-10 d incubation at the temperatures indicated. Leaf tissues for RNAseq analysis were excised at 24 and 48 hours after inoculation, snap frozen in liquid N_2_ and stored at -80^o^C for RNA isolation. Fungal culture samples were generated by inoculation of 50 ml of YPD broth, and growth to mid-logarithmic phase for 96h at 16C and 150 rpm. Cells were collected by vacuum filtration and snap frozen in liquid N_2_ and stored at -80^o^C for RNA isolation. Light microscopy of fungal hyphae growing on solid agar was performed using a Zeiss inverted light microscope.

**RNA isolation and Real-Time RT-PCR with Sybr-Green detection**

Total RNA was used for all Real-Time RT-PCR analyses. First-strand cDNA was synthesized from total RNA using the SuperScript III First Strand Synthesis System for RT-PCR (Invitrogen). A 5μg aliquot of total RNA primed with oligo(dT)_20_ was used in a 20μl reaction, following the supplier’s instructions. The resulting cDNA was analysed using a QuantiTect SYBR Green PCR Kit (QIAGEN), following the supplier’s instruction. The PCR reactions were run and analysed using an ABI 7500 Real Time PCR System. For expression normalisation, the beta-tubulin genes were used for *Z. tritici* *[1, 2]*

**Protein isolation and western blot analysis**

Anti-ZtGT2 polyclonal peptide antibodies were raised against KLH-coupled synthetic peptide sequence N-TWGSRDGADSSELNR-C by Eurogentec (Serraing Belgium). For Western blotting fungal protein extracts were generated as follows. Frozen fungal cells (~ 200 mg) were ground to fine powder in a mortar with liquid nitrogen. Three successive rounds of homogenization were used and material was checked for cell disruption after the third round under a light microscope. Frozen powder was then re-suspended in 5 ml of extraction buffer (50 mM Hepes pH 7; 0.25M sucrose + complete protease inhibitor cocktail (Sigma, St Louis)). Samples were mixed at 4C for 20 min on a rotating wheal mixer. Cell wall and debris were then pelleted by centrifugation at 1000g for 5 min. The supernatant was transferred into clean tubes and the pellet was washed twice by repeat centrifugation in extraction buffer as above. Finally, the pellet was solubilized by the addition of 400 μl of 2X SDS-PAGE loading buffer [3]. The supernatants were subjected to ultra-centrifugation in a Beckman Optima XL90 ultracentrifuge for 90 min at 70,000g and 4C. The soluble fraction was precipitated with the addition of 4 volumes of ice cold acetone at -20C for 2 hours, and the final pellet was suspended in 100 μl SDS-PAGE loading buffer. The pellet was washed once by repeat ultra-centrifugation in extraction buffer then res-suspended in 200 μl of loading buffer for SDS-PAGE and western blotting. Blotting was performed using the Biorad Mini-protean^TM^ system with nitrocellulose membranes subsequently washed and blocked in TBS-Tween + 5% skimmed milk powder. Affinity purified primary antibodies were used ta dilution of 1:200 in blocking reagent with anti-IgG-HRP as secondary antibody (1:10,000 Sigma St Louis). Blots were developed using ECL chemiluminescence (GE Healthcare) and visualized on a Li-cor Odyssey Fc scanner. Membranes were subsequently stained with Ponceau S for protein loading.

**Calcoflour white staining of fungal spores**

Plate grown *Z. tritici* (YPD) were stained for 5 mins with 0.001mg/ml Calcofluor white (Sigma: Fluorescent Brightener 28, catalogue no. F3543) in distilled water. A 10µl drop of the stained suspension was mounted on a glass slide with a cover slip. The slide was observed with a 40x objective lens using a Zeiss LSM 780 Confocal microscope (Carl Zeiss Ltd. Cambridge, UK), at 405nm excitation and 410 to 500nm emission.

**CFU based assay for alterations in leaf surface adhesion.**

We developed an assay which could overcome difficulties in maintaining sterility and identifying *Z. tritici* spores from other debris which could be released from washes of inoculated leaf surfaces. The assay makes use of the yeast-like colony growth form of *Z. tritici* on rich medium, and the drug selectable marker genes for G418 resistance that are present in both the WT Ku70 strain and the ∆ZtGT2 mutants generated in the Ku70 strain.

Spore suspensions of both strains were generated direct from 5 day old YPD agar plates in sterile water + 0.01% Silwet L-77 Silicone Surfactant. Spore suspensions were adjusted to 1 x10^5^ spores / ml as stocks. 100 µL of this stock suspension was pipetted onto (a) a glass slide to which spores should not adhere; and (2) onto the surface of a 5 cm long excised leaf fragment from wheat cultivar Riband. Materials were left for 30 mins to allow spore adhesion. Then each slide / leaf segment was carefully places into a 50 ml Falcon tube and was vortexed for 30s in the presence of 1 ml water + 0.01% Silvet. As a control the original stock spore suspensions were diluted 1 in 10 with the same solution and vortexed. Finally, 7.5 µL of the resulting aqueous solutions were each plated out onto a YPD agar plate containing 100 µg/ ml G418 and colony growth was allowed to proceed for 7 days. At this point the number of colonies retrieved was counted in segments from each plate. A total number of 20 replicates were performed for each treatment and the final values plotted along with their standard deviation as a percentage of the colonies obtained from the diluted stock spore suspension. Lower retrievable CFU numbers were indicative of greater adhesion to the surface in question.

**Assays for adhesion to hydrophobic (plastic) surfaces**

Spore suspensions at 1 X 10^4^ spores / ml were generated in sterile water + 0.01% tween 20. 400 µL was then pipetted into a single well of a 25 well polystyrene plate (Sterilin, Staffs UK). The plate was then left for 2 hours to allow spores to settle and adhere to the plastic surface. Then the liquid was removed from each well with a pipette and the underside of the well was marked to as to allow for imaging of the same region following the subsequent washes. Photomicrographs were taken for each marked region at this stage and then subsequently after 3 x successive 1ml washes with PBS. The photomicrographs were then used to count the number of cells in each image both prior to and after washes. Data were finally produced combining 5 replicate wells and a total of 10 images for each strain. The relative adhesion rate was presented as a percentage of the number of adhered cells prior to washing.

**Cell wall and culture filtrate monosaccharide analysis**

AIR (alcohol insoluble residue) of fungal cell walls or culture filtrates from both ∆ZtGT2 and WT strains grown in modified Brian medium [4] were analysed. Cultures were grown at 25 ^o^C for 5 days. Fungal cellular material was separated from the culture filtrate by filtration. For analysis of cell wall AIR monosaccharides, samples were processed by HPAEC-PAD as described in [5]. For analysis of culture filtrates, 120 ml of filtrate was precipitated with the addition of 2.5 vol of ice cold ethanol at 4 ^o^C overnight. Samples were collected by centrifugation, washed once in sterile water to remove some salts and then with 70% EtOH. Samples were then freeze dried. The equivalent of 100µg of material was used for the hydrolysis following homogenisation in water using a glass Teflon homogeniser and drying down prior to hydrolysis. Monosaccharides were then subsequently analysed and quantified as described in [5] above. Data was presented as mg /g dry weight from three biological replicate samples.

***F. graminearum* cellophane penetration assays for invasive growth**

Invasion assays on cellophane membranes (80 mm diameter, A.A. Packaging Limited) were performed as described [6]. A nitrogen response pathway regulates virulence functions in Fusarium oxysporum via the protein kinase TOR and the bZIP protein MeaB. Plant Cell 22, 2459-2475) with slight modifications. Cellophane disks were pre-soaked in sterile water and placed on potato dextrose agar (Sigma-Aldrich, UK). Eight μl water containing 200 *F. graminearum* conidia were plated onto the cellophane disks with a spreader and incubated at 22 C. After two days, cellophane disks with growing mycelium were removed. Plates were then incubated for further two days and fungal growth was photographed. Experiments were done three times with similar results.

***Supplementary references for methods***

1. Keon, J., et al., *Transcriptional adaptation of Mycosphaerella graminicola to programmed cell death (PCD) of its susceptible wheat host.* Molecular Plant-Microbe Interactions, 2007. **20**(2): p. 178-193.

2. Rudd, J.J., et al., *Identification and characterisation of Mycosphaerella graminicola secreted or surface-associated proteins with variable intragenic coding repeats.* Fungal Genetics and Biology, 2010. **47**(1): p. 19-32.

3. Laemmli, U.K., *CLEAVAGE OF STRUCTURAL PROTEINS DURING ASSEMBLY OF HEAD OF BACTERIOPHAGE-T4.* Nature, 1970. **227**(5259): p. 680-&.

4. Gravelat, F.N., et al., *Aspergillus Galactosaminogalactan Mediates Adherence to Host Constituents and Conceals Hyphal beta-Glucan from the Immune System.* Plos Pathogens, 2013. **9**(8).

5. Goubet, F., et al., *Cell wall glucomannan in Arabidopsis is synthesised by CSLA glycosyltransferases, and influences the progression of embryogenesis.* Plant Journal, 2009. **60**(3): p. 527-538.

6. Lopez-Berges, M.S., et al., *A Nitrogen Response Pathway Regulates Virulence Functions in Fusarium oxysporum via the Protein Kinase TOR and the bZIP Protein MeaB.* Plant Cell, 2010. **22**(7): p. 2459-2475.
